# Spectral-acoustic-coordinated astigmatic metalens for wide field-of-view and high spatiotemporal resolution 3D imaging

**DOI:** 10.1038/s41377-025-02180-7

**Published:** 2026-01-23

**Authors:** Shujian Gong, Yinghui Guo, Xiaoyin Li, Mingbo Pu, Peng Tian, Qi Zhang, Lianwei Chen, Wenyi Ye, Heping Liu, Fei Zhang, Mingfeng Xu, Xiangang Luo

**Affiliations:** 1 State Key Laboratory of Optical Field Manipulation Science and Technology, Institute of Optics and Electronics, Chinese Academy of Sciences, Chengdu, 610209 China; 2 Research Center on Vector Optical Fields, Institute of Optics and Electronics, Chinese Academy of Sciences, Chengdu, 610209 China; 3 College of Materials Sciences and Opto-Electronic Technology, University of Chinese Academy of Sciences, 100049 Beijing, China; 4 Sichuan Provincial Engineering Research Center of Digital Materials, Chengdu, 610213 China; 5 Tianfu Xinglong Lake Laboratory, Chengdu, 610213 China

**Keywords:** Metamaterials, Imaging and sensing

## Abstract

Metasurface-based light detection and ranging (LiDAR) is essential for high spatiotemporal resolution three-dimensional (3D) imaging in robotic and autonomous systems. Recent advances in inertia-free scanning techniques—such as acousto-optic and spectral scanning—have propelled the field forward. Nevertheless, key spatiotemporal metrics, including point acquisition rate (PAR), field-of-view (FOV), and imaging resolution, remain fundamentally constrained. These challenges are particularly acute in dual-axis LiDARs, where inter-axis rate mismatch and beam astigmatism degrade temporal and spatial resolution, respectively. Here, we present a wide-FOV, high spatiotemporal resolution LiDAR architecture with astigmatic metalens (AML) coordinated spectral-acousto-optic scanning. Consequently, a frame-wise point acquisition rate (FPAR) of 36.6 MHz (∼5-fold improvement over existing reports) and a wide FOV of 102° are simultaneously achieved. This breakthrough redefines LiDAR’s potential for ultra-high-speed, high-precision perception, enhancing applications such as autonomous driving with improved obstacle detection and safety at high speeds.

## Abstract (web summary)

This work presents a wide-FOV, high-spatiotemporal-resolution LiDAR with astigmatic metalens (AML) coordinated spectral-acousto-optic scanning. A frame-**wise** point acquisition rate of 36.6 MHz and a FOV of 102° are simultaneously achieved.

## Graphical abstract

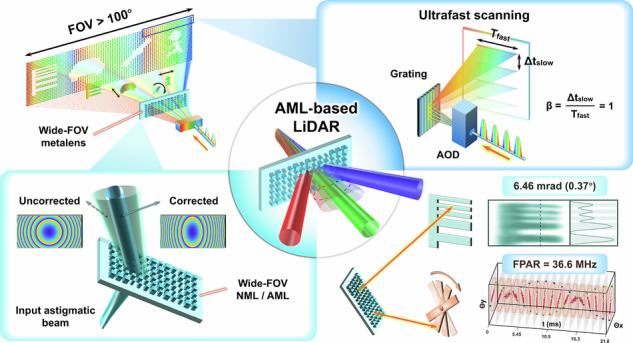

## Introduction

High spatiotemporal resolution three-dimensional (3D) imaging is pivotal for emerging applications in unmanned aircraft, autonomous vehicles, and robotics. While structured light imaging^1,2^ and stereovision^3,4^ provide 3D sensing capabilities, light detection and ranging (LiDAR) stands out due to its superior detection distance, precision, and environmental robustness^5–9^. Advanced LiDARs demand both high temporal resolution and spatial detection capabilities, necessitating two-dimensional (2D) beam scanning with rapid point acquisition rate (PAR), wide field-of-view (FOV), and dense resolvable points to capture dynamic objects with subtle changes. However, despite advancements in various scanning technologies, achieving wide-FOV, high spatiotemporal resolution simultaneously remains a critical challenge.

Traditional mechanical scanners, limited by bulk and instability, have spurred the development of compact, solid-state (inertial-free) alternatives^8^. Spatial light modulators (SLMs) achieve ~kHz PAR via liquid crystal reorientation^10^, while optical phased arrays (OPAs) reach ~MHz PAR but suffer from spatial sidelobes and fragility at high peak power^6,11^. Acousto-optic deflectors (AODs) cap scanning speeds due to acousto-optic transit time^12,13^ ( < 6 MHz to avoid beam distortion^14^). These conventional scanners are fundamentally limited by their low temporal resolution ceiling and an inherent trade-off between PAR and FOV: Inertial scanners provide wide FOVs at low PAR, while inertial-free scanners offer high PAR but narrow FOVs (e.g., SLMs: ~20°; AODs: ~2°).

In contrast, spectral scanning, enabled by intrinsically modulating the laser source through photonic time-stretching^15–18^ or time-frequency multiplexing, introduces a new paradigm for high-speed LiDAR, enabling inertia-free line scanning with PARs up to tens of MHz^17^. Yet, it still struggles to deliver 2D wide-FOV, high spatiotemporal resolution scanning. Firstly, the overall temporal resolution is restricted by the rate mismatch between fast and slow axis scanners, leading to slower imaging frame rates^19^. While spectral scanning significantly enhances the pixel-wise PAR (PPAR), the frame-wise PAR (FPAR) remains bottlenecked by the mechanical slow axis^7,17,20–22^, where FPAR represents the effective PAR accounting for duplicate points during full-frame scanning. Secondly, the scanning FOV is inherently constrained by both the available spectral bandwidth and the dispersion capabilities of common gratings^23^, limiting FOV to only a few degrees. Expanding the FOV may introduce additional distortions in cascaded dual-axis scanning systems, where the non-coplanar scanners induce beam deflection dependencies^24,25^. A common mitigation strategy employs a 4 f system for optical conjugation and center pivot alignment^26^, widely adopted in cascading galvanometers, AODs, and SLMs across microscopy^26,27^, multi-beam optics^24^, and laser processing^28^; however, it inevitably increases system complexity and volume. Thirdly, the spatial resolution is degraded by beam astigmatism arising from the intrinsic anisotropy of diffractive gratings. Blazed gratings disperse light in one direction while reflecting it in the orthogonal direction, inducing asymmetric angular divergence. This anisotropy induces astigmatism, distorting the beam into an undesirable elliptical profile and ultimately degrading the spatial resolution. Except for specialized applications such as axial positioning in microscopy imaging^29,30^, astigmatism is generally undesirable^31^. Conventional optical designs typically rely on bulky cylindrical lenses or meticulously engineered GRIN lenses to mitigate astigmatic effects^32^.

Over the past decade, metasurfaces^33–36^ have captivated the photonics community by enabling precise control over amplitude, phase, frequency, polarization, and more. They offer solutions to many challenges faced by conventional optics, ushering in Optical Engineering 2.0^37^. The multidimensional optical field manipulation capabilities of metalenses (MLs) enable the on-demand design of point spread function (PSF) and allow for functions such as momentum transformation, spectral focus tuning, and astigmatism correction. These advancements have led to widespread applications in angular momentum detection^34^, spectral tomographic imaging^38^, quantitative phase imaging^39^, and spectroscopy^40^. In passive imaging, a quadratic phase metalens based on symmetry transformation^41^ can achieve a wide FOV of ±80°, yet it has not been applied to active laser imaging with single-pixel detection. Recently, in active imaging, Ref^14^. addressed the limited FOV in MHz-level homogeneous dual-axis AOD scanning by cascading a metasurface. However, its simple circularly symmetric phase distribution also leads to FOV distortion, increased beam divergence, and degraded spatial resolution.

Here, we propose a wide-FOV, high spatiotemporal resolution LiDAR architecture based on an astigmatic metalens (AML), which integrates solutions to the challenges mentioned above. To enhance temporal resolution, we employ spectral scanning (PPAR ~tens of MHz) for the fast axis and AOD (several MHz) for the slow axis, achieving inter-axis “rate matching” and significantly improving the final FPAR. To expand the spatial detection capabilities of spectral-acousto-optic (spectral-AO) dual-axis cascade scanning, we have uniquely designed an AML that enlarges the FOV while maintaining a small divergence angle through astigmatism correction. As a result, we demonstrate an advanced LiDAR architecture with both PPAR and FPAR reaching 36.6 MHz ( ~ 5-fold improvement over existing reports), ensuring high temporal resolution, while also attaining a spatial resolution of 0.37° within a wide 102° FOV. Further, we also achieve an ultra-high-speed imaging frame rate of 20.3 kfps with 1800 points per frame. The achieved FPAR enables mega-pixel 3D imaging at video frame rates, making it highly promising for broader applications that demand dynamic high-precision perception, such as autonomous driving and high-speed small drone tracking.

## Results

### Principle of wide-FOV high spatiotemporal resolution LiDAR

**Fig. 1:**
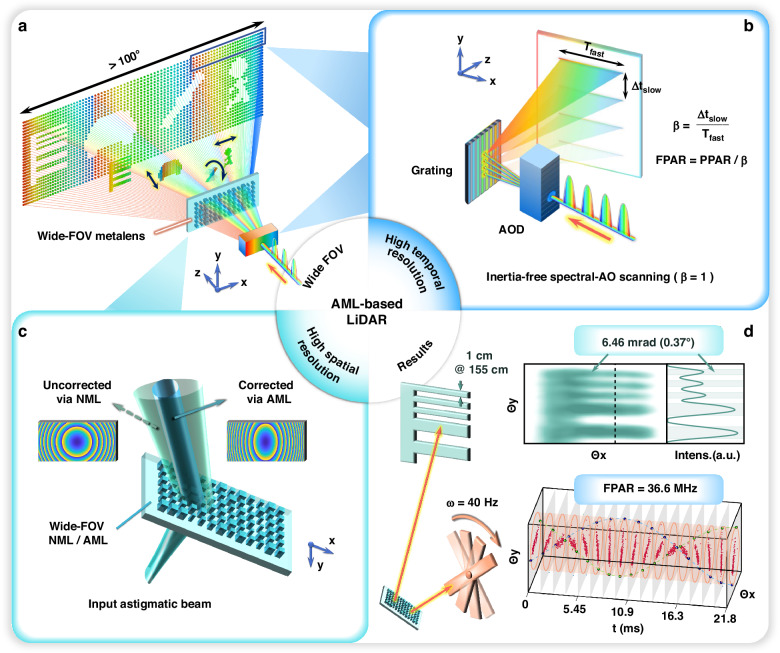
Concept of the wide-FOV, high spatiotemporal resolution LiDAR. **a** Proposed LiDAR architecture integrating a metalens for wide-FOV capture of subtle environmental dynamics. **b** Implementation of high temporal resolution. Inter-axis rate matching via spectral-acousto-optic (spectral-AO) scanning enables full-field high-speed two-dimensional beam scanning. **c** Achievement of high spatial resolution. The wide-FOV astigmatic metalens (AML) simultaneously addresses the challenges of narrow spectral scanning FOV and beam astigmatism that degrade spatial resolution—issues that cannot be mitigated by a normal metalens (NML) without astigmatic compensation. Insets show the schematic phase profiles of the NML and AML. **d** Top: Imaging of a resolution target, demonstrating the system’s superior spatial resolution (6.46 mrad). Bottom: Time-sliced evolution of a rapidly rotating fan, highlighting the system’s high temporal resolution (FPAR ~ 36.6 MHz)

The spatiotemporal sensing capability of a LiDAR system is primarily defined by its temporal resolution and spatial detection capability, enabling the capture of rapid and subtle environmental changes across a wide FOV, as shown in Fig. 1a.

On the one hand, LiDAR’s temporal resolution is determined by the PAR of its scanning mechanism. LiDAR operates by modulating the transmitted laser waveform and measuring the round-trip time-of-flight (TOF) *τ* of photons^42^. In traditional single-pulse direct-TOF LiDAR systems, the PPAR is equal to the laser repetition rate *f*_rep_. Here, by employing time-frequency multiplexing, a broadband laser pulse is modulated into a discrete sequence of chirped pulses across *N*_*λ*_ spectral channels, thereby enhancing the PPAR accordingly:

1 $${\rm{PPAR}}={N}_{\lambda }\cdot {f}_{{\rm{rep}}}$$

As illustrated in Fig. 1b, spectral scanning is achieved through grating dispersion of the *N*_*λ*_ channels, providing a high PPAR (up to tens of MHz) and serving as the fast axis. However, if the point-switching rate of the slow axis lags behind the line rate of the fast axis, causing the fast axis to redundantly scan the same region *β* times, the effective FPAR will be reduced by a factor of *β* relative to the PPAR:

2 $$\beta =\frac{{\mathrm{fast}}\,{\mathrm{axis}}\,{\mathrm{line}}\,{\mathrm{rate}}}{{\mathrm{slow}}\,{\mathrm{axis}}\,{\mathrm{point}}\,{\mathrm{rate}}}=\frac{\Delta {t}_{{\mathrm{slow}}}}{{T}_{{\mathrm{fast}}}}$$

3 $${\rm{FPAR}}={\rm{PPAR}}/\left(\mathop{\prod }\limits_{i=1}^{{n}_{{\rm{a}}}-1}{\beta }_{i}\right)$$

where *β*_*i*_ denotes the inter-axis rate mismatch factor (RMF) arising from the transition between the *i*-th and (*i* + 1)-th scanning axes (total *n*_a_ axes). PPAR is the instantaneous PAR determined by the fastest scanning axis, while FPAR represents the effective PAR during full-frame scanning, accounting for duplicate points and further restricted by the slow axis. To ensure that the FPAR reaches the maximum PPAR attainable through spectral scanning, the slow axis switching must be synchronized with the line rate of the fast axis spectral scanning, corresponding to *f*_rep_. To achieve this, we introduce an AOD with a point-switching rate of up to MHz-level (matching *f*_rep_) for the slow axis, ensuring perfect inter-axis “rate matching” with *β* = 1 and thus aligning FPAR with the maximum PPAR.

On the other hand, the spatial detection capability (***C***_spatial_) of LiDAR reflects its ability to resolve small objects over a wide FOV and is expressed as:

4 $${{\boldsymbol{C}}}_{{\rm{spatial}}}={N}_{x}\cdot {N}_{y}\cdot FO{V}_{x}\cdot FO{V}_{y}=\frac{{(FO{V}_{x}\cdot FO{V}_{y})}^{2}}{{\theta }_{x}\cdot {\theta }_{y}}$$

where *FOV*_*x*_ and *FOV*_*y*_ represent the horizontal and vertical scanning FOVs, respectively, while *N*_*x*_ and *N*_*y*_ denote the effective number of resolvable points, defined as the ratio of the FOV to the angular resolution *θ* in the corresponding direction.

Therefore, achieving minimal angular divergence over a wide FOV is crucial for enhancing spatial detection capability. While conventional telescope systems can expand the narrow FOV of spectral scanning, they fail to compensate for the degradation in spatial resolution caused by beam astigmatism. Here, we introduce a wide-FOV AML that simultaneously addresses the challenges of narrow FOV and beam astigmatism—issues that cannot be mitigated by a normal metalens (NML) without astigmatic compensation, as illustrated in Fig. 1c.

In summary, we employ a wide-FOV AML to harmonize spectral-AO cascade scanning, achieving exceptional comprehensive spatiotemporal resolution, as illustrated in Fig. 1d. The top panel shows the imaging results of a resolution target, where *Θ*_*x*_ and *Θ*_*y*_ denote the object’s angular position relative to the AML. The shaded region in the accompanying Intensity-*Θ*_*y*_ map highlights the angular range containing real features, demonstrating the ability to resolve a 1 cm-wide feature at a distance of 155 cm, corresponding to a spatial resolution of 6.46 mrad. The bottom panel illustrates dynamic imaging of a high-speed rotating fan, with each slice representing a single frame (frame rate: 734 fps; 600 × 83 points per frame). The results reveal a temporal resolution corresponding to an FPAR of 36.6 MHz. This comprehensive spatiotemporal resolution outperforms previously reported LiDARs in comparable settings (see Supplementary Fig. S1 and Table S1 for detailed comparisons).

**Fig. 2:**
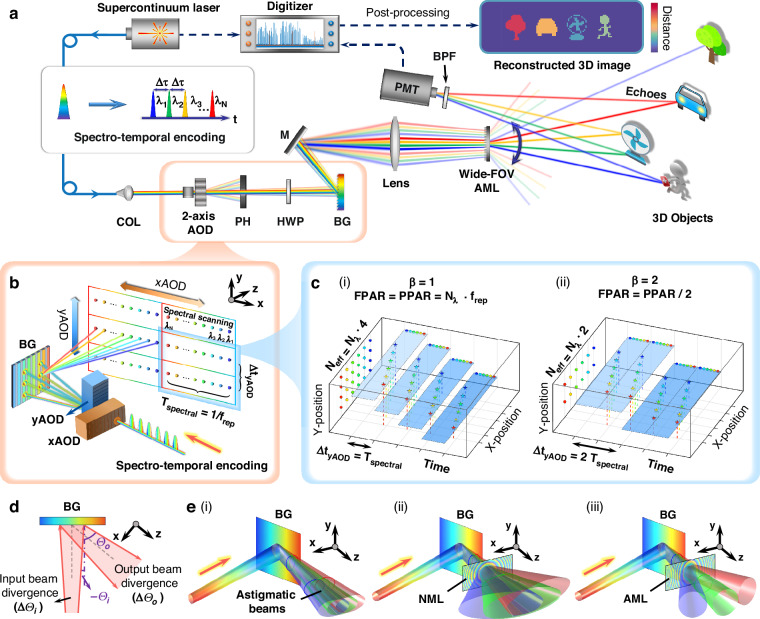
Implementation of the wide-FOV high spatiotemporal resolution LiDAR. **a** Experimental setup. A broadband source undergoes spectro-temporal encoding for time-frequency multiplexing. The resulting discrete chirped sub-pulses are directed through a 2-axis AOD and a blazed grating (BG) for spectral-dual-AO scanning. The beam then passes through the wide-FOV astigmatic metalens (AML) to enhance spatial detection capability. Echoes are collected by a photomultiplier tube (PMT) for 3D reconstruction. COL, collimating lens; PH, pinhole; HWP, half-wave plate; M, mirror; BPF, bandpass filter. **b** Schematic of spectral-dual-AO cascade scanning. The *y*AOD swiftly transitions after each spectral scan to ensure rate matching, while the *x*AOD operates similarly, forming a three-axis scanning configuration. **c** Impact of rate mismatch on the effective number of acquired points. (i) Rate matching (*β* = 1) ensures FPAR = PPAR, maximizing acquisition efficiency; (ii) Rate mismatch (*β* = 2) reduces FPAR to half of PPAR due to redundant spectral scans. **d** Schematic of beam divergence angle expansion induced by the BG. **e** Schematic of the output beams evolution after diffraction by the BG for 3 adjacent spectral channels: (i) without ML, (ii) with NML, and (iii) with AML. The AML corrects beam astigmatism while simultaneously expanding the spectral scanning FOV

The implementation of the proposed wide-FOV high spatiotemporal resolution LiDAR system is provided in Fig. 2. As depicted in Fig. 2a, the broad-spectrum pulsed light from a supercontinuum laser undergoes spectro-temporal encoding, converting it into a discrete chirped pulse sequence with uniform temporal and spectral intervals (details in Supplementary Section 2). The encoded light then propagates through a dual-axis AOD, enabling small-angle 2D beam scanning. It then traverses a blazed grating (BG) to implement spectral-AO dual-axis scanning. To address the narrow FOV and the beam astigmatism induced by the BG’s anisotropic properties, we incorporate a Galileo-like telescope system composed of an AML at the backend, effectively reducing beam divergence while expanding the FOV. The echo signals are detected by a single-pixel photomultiplier tube (PMT), while the laser pulse serves as a reference to extract TOF information, ultimately enabling the 3D reconstruction of target scene.

Through discrete time-stretching^17^, the spectro-temporal encoding module achieves *N*_*λ*_-channel time-frequency mapping (Supplementary Fig. S2d). According to Eq. (1), a PPAR of 36.6 MHz is achieved by *N*_*λ*_ = 30 spectral channels with *f*_rep_ = 1.22 MHz. We introduce AODs to match this fast axis line rate, as illustrated in Fig. 2d. Given that the point-switching time of *y*AOD is Δ*t*_*y*AOD_, and assuming the slower *x*AOD aligns with one complete *y*AOD scanning period (*β*_2_ = 1), the effective FPAR can be calculated as follows:

5 $$\mathrm{FPAR}=\mathrm{PPAR}/({\beta }_{1}\cdot {\beta }_{2})={N}_{\lambda }\cdot {f}_{\mathrm{rep}}/\left(\frac{\Delta {t}_{y\mathrm{AOD}}}{{T}_{\mathrm{spectral}}}\cdot 1\right)=\frac{{N}_{\lambda }}{\Delta {t}_{y\mathrm{AOD}}}$$

where *T*_spectral_ = 1/*f*_rep_ is the period of spectral scanning. Thus, FPAR depends on the point-switching time of *y*AOD, which corresponds to the slow axis of spectral scanning. If Δ*t*_*y*AOD_ = *T*_spectral_, the system ensures seamless transitions between positions after each spectral scan, maintaining *β*_1_ = 1 and preventing redundant scans. In this case, FPAR reaches its maximum value (equal to PPAR), enabling a high-speed three-axis scanning format known as spectral-dual-AO cascade scanning, as illustrated in Fig. 2c(i), with the scanning timing diagram provided in Supplementary Fig. S3. In contrast, if Δ*t*_*y*AOD_ > *T*_spectral_, FPAR falls below PPAR due to repeated spectral scanning, as illustrated in Fig. 2c(ii). This highlights the fundamental limitation of full-field imaging rates in conventional spectral scanning LiDARs that rely on bulky mechanical scanning as the slow axis.

High temporal resolution alone is insufficient for an exceptional LiDAR system, as wide detection FOV and high angular resolution are equally crucial. In addition to the difficulty in achieving inter-axis rate matching, another major drawback of spectral scanning is its restricted spatial detection capability—both the narrow FOV and degraded spatial resolution induced by beam astigmatism. According to the grating equation:

6 $${{\varTheta }}_{o}=\arcsin \left(\frac{\lambda }{d}-\,\sin \,{{\varTheta }}_{i}\right)$$

where *Θ*_*i*_ and *Θ*_*o*_ represent the incidence and diffraction angles of the blazed grating, respectively (same sign on the same side of the normal); *λ* denotes the incident wavelength (centered at 1547.5 nm with a bandwidth of 13 nm); and *d* is the grating constant (1/600 mm). Although increasing the wavelength bandwidth and grating density can enlarge the FOV, it remains constrained to only a few degrees. Conventional telescopes can extend the FOV but introduce mismatches in cascaded dual-axis scanning systems. The inevitable spatial separation of the two scanners causes beam deflection along the second scanning axis to depend on the first axis^24,25^, thereby exacerbating distortions during FOV expansion. Moreover, increased third-order (Seidel) aberrations—such as astigmatism and coma—further constrain the FOV^43^. Specifically, in spectral scanning, beam astigmatism primarily arises from the grating’s anisotropy, which induces additional angular divergence in the dispersion direction (effectively reducing the virtual focal length). A differential analysis yields:

7 $$\mathop{\mathrm{lim}}\limits_{{\it\varTheta }_{i}\to 0}\frac{d{\it\varTheta }_{o}}{d{\it\varTheta }_{i}}=\mathop{\mathrm{lim}}\limits_{{\it\varTheta }_{i}\to 0}\frac{-\,\cos \,{\it\varTheta }_{i}}{\sqrt{1-{(\frac{\lambda }{d}-\,\sin \,{\it\varTheta }_{i})}^{2}}}=\frac{-1}{\sqrt{1-{(\frac{\lambda }{d})}^{2}}}=\frac{-1}{\sqrt{1-{\sin }^{2}{\it\varTheta }_{o}}}|_{{\it\varTheta }_{i}=0}=\frac{-1}{\cos \,{\it\varTheta }_{o}}|_{{\it\varTheta }_{i}=0}$$

This reveals that the BG expands the output angular range (divergence angle) by a factor of cos^-1^*Θ*_*o*_ relative to the input, as illustrated in Fig. 2d. From a physical optics perspective, this effect stems from the large-angle wavevector deflection imparted by the BG, which reduces the effective beam waist size in the propagation direction by a factor of cos *Θ*_*o*_. According to Gaussian beam theory, a smaller beam waist results in a larger divergence angle. Calculations indicate that for monochromatic incident light at *λ* = 1550 nm, the divergence angle of the diffracted beam along the grating’s dispersion direction increases by ~2.72 times, while in the orthogonal direction, the grating acts as a planar mirror, contributing no divergence expansion. This anisotropic effect leads to beam astigmatism, causing the output beam to evolve from elliptical to circular and back to elliptical along the optical axis, as depicted in Fig. 2e(i).

Applying an NML with non-astigmatic, radially symmetric phase can expand the FOV but fails to mitigate astigmatism. The divergence angle increases synchronously with the FOV magnification in both orthogonal directions, preserving the beam’s astigmatic characteristics and limiting the resolution for adjacent spectral channels, as shown in Fig. 2e(ii).

The metalens’s capability to arbitrarily manipulate wavefronts offers a novel solution. By engineering an astigmatic phase profile with distinct focal lengths for tangential and sagittal rays, the AML can effectively correct anisotropy-induced astigmatism. This results in more uniform divergence angles in both directions and a nearly circular beam profile, significantly suppressing astigmatism across a wide FOV and thereby greatly enhancing the spatial detection capability of spectral scanning, as illustrated in Fig. 2e(iii). (In actual experiments, a lens is used to focus the beam onto the AML.)

### Wide-FOV astigmatic metalens enabled high spatial detection capability

Previously, we introduced a wide-FOV AML to enhance the spatial detection capability of spectral scanning, addressing both its limited FOV and beam astigmatism. In addition, FOV mismatching, a critical issue in heterogeneous dual-axis cascade scanning, manifests in two key forms—FOV distortion and a rectangular output FOV. FOV distortion arises from the unavoidable spatial separation between the two orthogonal scanners, which causes beam deflection by the second scanner to be dependent on the first. Meanwhile, in heterogeneous cascade scanning, FOV mismatching also manifests as inconsistent scanning FOVs along the two axes, resulting in a rectangular output FOV. When a conventional telescope is introduced for FOV expansion, both forms of FOV mismatching are further exacerbated, amplifying distortion and degrading overall performance.

**Fig. 3:**
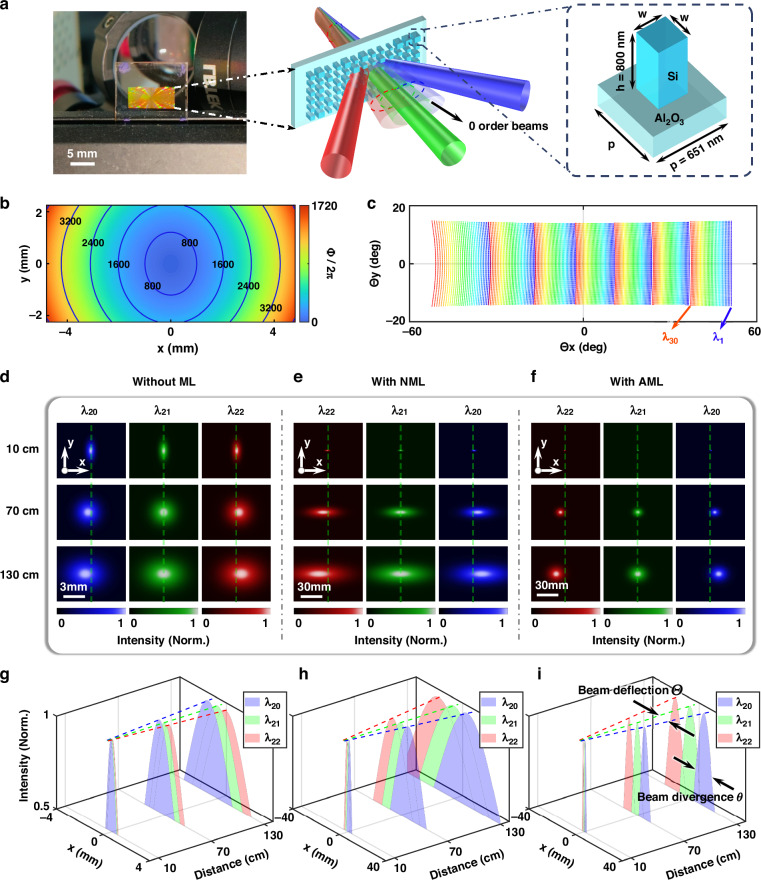
Enhancement of LiDAR spatial detection capability enabled by the wide-FOV AML. **a** Schematic illustration of the wide-FOV AML-enhanced spatial detection mechanism. Compared to unmodulated 0-order beams, the AML both expands the FOV and improves spatial resolution. Left panel: photograph of the fabricated AML. Right panel: schematic of the meta-atom unit cell composing the AML. **b** Phase distribution of the wide-FOV AML, with gradient colors representing the phase and blue contour lines indicating the phase gradient (rad/mm). **c** Output angular distribution of the 2D scanning beams enabled by spectral-AO cascade scanning assisted by the AML, referenced to 0° at the beams’ center, with colors representing the spectral channels. Evolution of three adjacent channel beams diffracted by the grating: **d** without ML, **e** with NML, and **f** with AML. The green dashed line marks the *x*-position of the middle-channel beam as a coordinate reference. Due to astigmatism, the grating output beams evolve from elliptical to circular and back to elliptical; this aberration is effectively corrected by the AML. Corresponding intensity profiles in the *y* = 0 plane: **g** without ML, **h** with NML, and **i** with AML. The AML expands the FOV, corrects astigmatism and improves spatial resolution, thereby markedly enhancing LiDAR’s spatial detection capability

We use commercial software (*Zemax OpticStudio*) to optimize the AML’s phase profile, effectively addressing the aforementioned issues while expanding a wide FOV and correcting beam astigmatism. The phase design draws inspiration from the symmetry transformation^41^ of the focusing quadratic phase (*Φ*(*r*) = -*k*_0_*r*^2^/(2*f*_eff_))—this transformation enables varying incident angles to induce lateral shifts of the focal spot, realizing an angle-to-position mapping, where *k*_0_ = 2π/*λ*, *f*_eff_ denotes the effective focal length, and *r* is the radial coordinate. Building on this, we innovatively adopt a divergent quadratic phase as the AML’s phase base to achieve inverse mapping between the incident position on the AML and the wide-FOV output angle; meanwhile, we incorporate higher-order astigmatic terms to resolve the inherent trade-off between wide FOV and high spatial resolution. The specific phase distribution of the AML is as follows:

8 $${{\varPhi }}_{{\rm{AML}}}(x,y)=\frac{{c}_{1}{x}^{2}+{c}_{2}{y}^{2}}{{r}_{m}^{2}}+\frac{{c}_{3}{x}^{4}+{c}_{4}{x}^{2}{y}^{2}+{c}_{5}{y}^{4}}{{r}_{m}^{4}}+\frac{{c}_{6}{x}^{6}+{c}_{7}{x}^{4}{y}^{2}+{c}_{8}{x}^{2}{y}^{4}+{c}_{9}{y}^{6}}{{r}_{m}^{6}}$$

where *x* and *y* represent the spatial coordinates on the metalens, and *r*_*m*_ = 5 mm is the radius of its effective region. The coefficients *c*_*i*_ (*i* = 1, 2, …, 9) corresponding to the phase terms of each order are listed in the Supplementary Table S2. Details on the AML’s optimization design are provided in Supplementary Section 4. This design not only expands the FOV but also enhances spatial resolution, as illustrated in Fig. 3a. The left panel presents a photograph of the fabricated AML, while the right panel illustrates the meta-atom unit cell structure of sapphire-on-silicon (SOS) (see Supplementary Fig. S4 for details). As depicted in Fig. 3b, the AML’s phase distribution exhibits an elliptical-like arrangement of isophase lines. Compared to conventional non-astigmatic phase profiles of the form *Φ*(*r*^2^), this tailored design introduces additional phase terms and degrees of freedom, mitigating FOV mismatching during rectangular FOV expansion while effectively suppressing beam divergence via astigmatism correction. The resulting wide-FOV output angle distribution, *Θ*_*x*,*y*_, is illustrated in Fig. 3c, where *Zemax* simulations indicate a total scanning FOV of 102° × 26°. The field distortion is well corrected in spherical coordinates, preserving an approximately rectangular output FOV. The slight horizontal asymmetry arises from the nonlinear relationship inherent in the grating equation Eq. (6). Details on the characterization and imaging experiments for the wide FOV are provided in Supplementary Section 5.

To characterize the enhancement in spatial detection capability provided by the wide-FOV AML, we measured the divergence angles of beams from three adjacent spectral channels (*λ*_20_, *λ*_21_, *λ*_22_) after grating diffraction, comparing cases with and without the metalens. The measured spot sizes at various distances and the divergence angle fitting processes are detailed in Supplementary Section 6. These data were used to model beam propagation at extended distances, as shown in Fig. 3d–i.

Specifically, Fig. 3d–f display the evolution of the beams at different exit distances for the three spectral channels after grating dispersion, (d) without ML, (e) with NML, and (f) with AML, respectively. The green dashed line represents the *x*-position of the beam at the wavelength *λ*_21_ (1550.1 nm) as the coordinate reference. Fig. 3d confirms that the grating output beams are astigmatic, evolving from elliptical to circular and back to elliptical. The divergence angle ratio between the two directions is 2.36 mrad / 0.80 mrad = 2.95, slightly exceeding the theoretical value of 2.72 calculated from Eq. (7), which supports the reliability of our experiment (with discrepancies primarily arising from the spectral bandwidth of a single incident channel). As shown in Fig. 3e, expanding the FOV using a non-astigmatic conventional telescope system does not eliminate beam astigmatism; instead, the divergence angle increases proportionally with the FOV angle, leaving the effective number of resolvable points unchanged. In contrast, Fig. 3f demonstrates that the AML effectively eliminates beam astigmatism. The astigmatic characteristics decouple the divergence angle from the FOV angle, enabling simultaneous FOV expansion and divergence suppression. This leads to a significant improvement in spatial resolution for adjacent channels compared to the NML, thereby increasing the effective number of resolvable points while simultaneously broadening the FOV. The output beam divergence angles in the two directions are *θ*_*x*_ = 11.8 mrad and *θ*_*y*_ = 10.2 mrad, which are largely consistent (see Supplementary Fig. S9 for details). Fig. 3g–i illustrate the evolution of horizontal beam intensity profiles at various exit distances for the three adjacent channels, without ML, with NML, and with AML, respectively.

Based on the measured divergence and deflection angles for these three-channel beams, calculations indicate that the AML not only expands the FOV but also enhances the effective resolvable points, significantly improving the spatial detection capability of the LiDAR system. As shown in Table 1, the overall ***C***_spatial_ is improved by two orders of magnitude compared to a system without the wide-FOV AML (see Supplementary Section 6 for details of the listed values).

**Table 1:** Comparisons of emission beam metrics under different conditions

Parameters	Notes	Without ML	With NML	With AML
$\overline{\varDelta {\it\varTheta }_{{x}\_{{\lambda }}_{{\boldsymbol{i}}}-{{\lambda }}_{{\boldsymbol{i}}+{\bf{1}}}}}(\mathrm{mrad})$	*x*-direction addressing angle ^a^	0.56	9.4	9.4
$\overline{{{\theta }}_{{x}}}(\mathrm{mrad})$	*x*-direction divergence angle ^b^	2.36	39.6	11.8
$\overline{{{\theta }}_{{y}}}(\mathrm{mrad})$	*y*-direction divergence angle ^b^	0.80	13.4	10.2
${{\boldsymbol{C}}}_{{\rm{spatial}}-{\rm{1D}}-{\lambda }_{20} \sim {\lambda }_{22}}(\mathrm{mrad})$	$\frac{{(2\cdot \overline{\varDelta {{\it\Theta }}_{{x}\_{{\lambda }}_{{\boldsymbol{i}}}-{{\lambda }}_{{\boldsymbol{i}}+{\bf{1}}}}})}^{2}}{\overline{{{\theta }}_{{x}}}}$	0.53	8.9	30.0
$FO{V}_{x}\cdot FO{V}_{y}$	FOV of full-field scanning beams ^c^	8.0° × 2.4°	102° × 31°	102° × 26°
${{\boldsymbol{C}}}_{{\rm{spatial}}-2{\rm{D}}-{\rm{total}}}(\mathrm{ra}\mathrm{d}^{2})$	$\frac{{(FO{V}_{x}\cdot FO{V}_{y})}^{2}}{\overline{{{\theta }}_{{\boldsymbol{x}}}}\cdot \overline{{{\theta }}_{{\boldsymbol{y}}}}}$	18.12	1748	5422

^a^The angle between the emission beams of adjacent channels

^b^Half-angle at which the intensity drops to 1/*e*² of its maximum

^c^Including all 30 channels acquired via spectral-dual-AO cascade scanning

### High temporal resolution dynamic 3D imaging

**Fig. 4:**
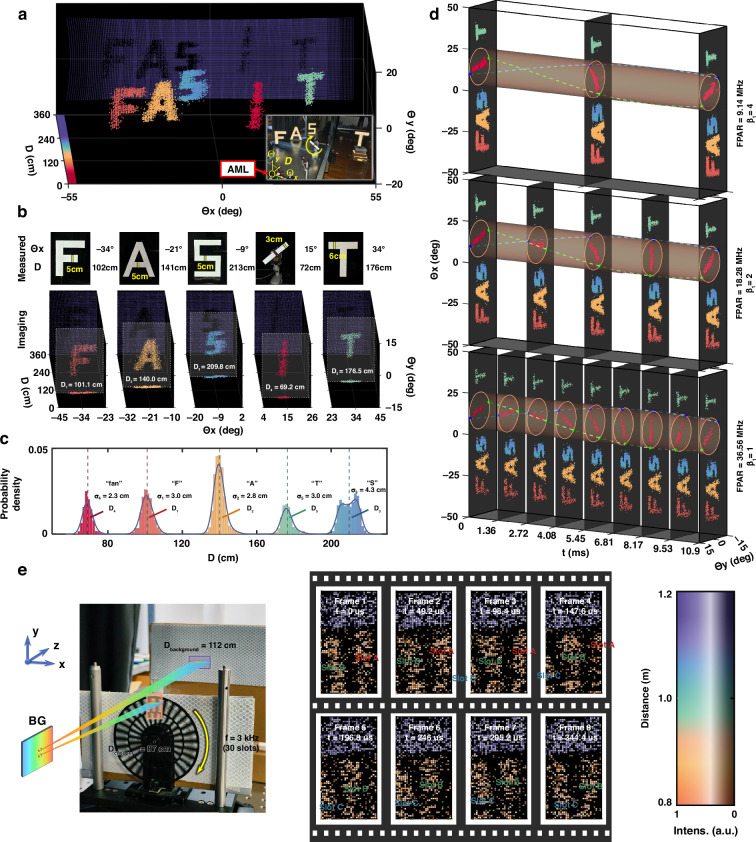
High temporal resolution 3D imaging. **a** 3D imaging results and the corresponding scene (inset) containing the letters “F”, “A”, “S”, “T”, and a rapidly rotating fan. The single-frame results provide a total of 20(*x*AOD)×83(*y*AOD)×30(spectral channels) addressable spatial points. **b** Measured object dimensions and spatial positions (top) compared with reconstructed 3D point clouds (bottom). **c** Probability density distribution of the point clouds along the distance axis, demonstrating a ranging precision of ~3 cm (mean of the standard deviation *σ*). **d** Temporal evolution of fan rotation at varying FPARs (maximum 36.56 MHz for *β*_1_ = 1). Orange cylinders and blue/green helices represent time-resolved fan states, with helical pitch analysis yielding a rotation period of 24.94 ms (fitted) versus 25.10 ms (ground truth). **e** Ultra-high-speed dynamic 3D imaging results at 20.3 kfps frame rate (1×60×30 addressable points per frame). The imaging results show that after 6 ~ 7 frames, Slot C rotates to the original position of Slot B, which is consistent with the single slot period of ~333.3 μs for a 3 kHz chopper

To demonstrate the high temporal resolution of our system, we conducted dynamic 3D imaging experiments on high-speed rotating fan blades. As illustrated in Fig. 4a, the imaging scene comprises the letters “F”, “A”, “S”, and “T” along with a rapidly spinning fan. The spherical coordinate system, centered at the AML, defines the axes as horizontal (azimuth) angle *Θ*_*x*_, vertical (elevation) angle *Θ*_*y*_, and radial distance *D*. The top panel of Fig. 4b specifies the dimensions of each object along with their coordinates (*Θ*_*x*_, *D*), with the objects’ edge horizontal FOV reaching ±40°. Detailed data processing procedures for obtaining the dynamic 3D point clouds are provided in Supplementary Section 7.

The achieved PPAR of 36.56 MHz is determined by the spectral scanning. According to Eq. (5), the full-field FPAR depends on *β*_1_, which represents the ratio of the *y*AOD point-switching time to the spectral scanning period. When *β*_1_ = 1, FPAR reaches its maximum (equal to PPAR). If *β*_1_ > 1, FPAR decreases to PPAR/*β*_1_; however, each *y*AOD scanning position will undergo *β*_1_ complete spectral scans, allowing for averaging of these data points to further mitigate noise. Accordingly, we conducted imaging experiments at *β*_1_ values of 4, 2, and 1.

In this experiment, we implement sub-pixel spectral-AO scanning with 20 scanning points provided by the *x*AOD and 83 points by the *y*AOD for finer resolution (detailed in Supplementary Section 3), resulting in *N*_*tot*_ = 20×83×30 addressable points (30 spectral channels). In the bottom panel of Fig. 4b, the 3D imaging results at *β*_1_ = 4 reveal that the letters “F”, “A”, “S”, and “T” as well as two fan blades are resolved with high fidelity. Here, with FPAR = PPAR/4 = 9.14 MHz, the system achieves an image frame rate of 183.5 fps (FPAR/*N*_*tot*_) and a per-frame acquisition time of 5.45 ms. Distance measurements—calculated as the mean ± standard deviation across the point clouds of each object—align with ground-truth values and demonstrate a ranging precision of ~3 cm, as shown in Fig. 4c. The dominant source of error arises from the inherent transit time spread (TTS) of the PMT (see Supplementary Section 8 for details).

For *β*_1_ = 2 and *β*_1_ = 1, FPAR values of 18.28 MHz and 36.56 MHz are achieved, with corresponding single-frame acquisition durations of 2.72 ms and 1.36 ms, resulting in frame rates of 367 fps and 734 fps, respectively. Time-slice diagrams depicting the front views of the 3D images acquired under these three *β*_1_ conditions are illustrated in Fig. 4d, with color indicating distance from the AML.

Although the *β*_1_ = 4 condition offers a higher number of effective points and improved imaging quality, its lower frame rate results in less clarity for capturing the fan’s motion. Moreover, when the fan is oriented horizontally, the slow scan speed in the *β*₁ = 4 group results in noticeable blade “deflection”, as shown in the time slice at *t* = 5.45 ms. Within the 5.45 ms duration of single-frame acquisition for *β*_1_ = 4, the *β*_1_ = 2 group captures 2 frames, while the *β*_1_ = 1 group captures 4 frames. As FPAR increases, the fan’s motion becomes more discernible, allowing us to model the evolution of the fan blade endpoints as a helical trajectory (with the blue and green spirals representing the endpoints’ motion). The projection of the helix onto the *t*-*Θ*_*y*_ plane forms a cosine function, yielding a measured fan rotation period of 24.94 ms, closely aligning with the actual value of 25.10 ms. This confirms the system’s capability for high temporal resolution, dense point-cloud 3D imaging across a wide FOV, and precise measurement of rotational speeds.

Furthermore, we reduced the number of scanning points to achieve a higher imaging frame rate of 20.3 kfps at *β*_1_ = 1, as shown in Fig. 4e. The wide-FOV AML was temporarily omitted while fixing the *x*AOD scanning frequency (see Supplementary Section 3.2 for details). The *y*AOD scanned 60 points vertically, yielding a total of 1×60×30 points. The middle panel of Fig. 4e presents 8 frames of imaging results, illustrating the continuous positional shifts of three adjacent slots within the scanning FOV. Between Frames 7 and 8, Slot C rotates to the original position of Slot B in Frame 1, demonstrating that 6 ~ 7 frames were captured within a 333.3 μs chopper period (operating at *ω* = 100 Hz with 30 slots). This validates the system’s ultra-high-speed 20.3 kfps 3D imaging frame rate (see Supplementary Movie S3 for 40 dynamic frames captured over a 2 ms recording duration).

### High spatial resolution 3D imaging assisted by subpixel reconstruction

The key advantage of the proposed LiDAR architecture is its ability to achieve both high temporal and spatial resolution. To evaluate its spatial resolution, we conducted another 3D imaging experiment using static resolution targets.

**Fig. 5:**
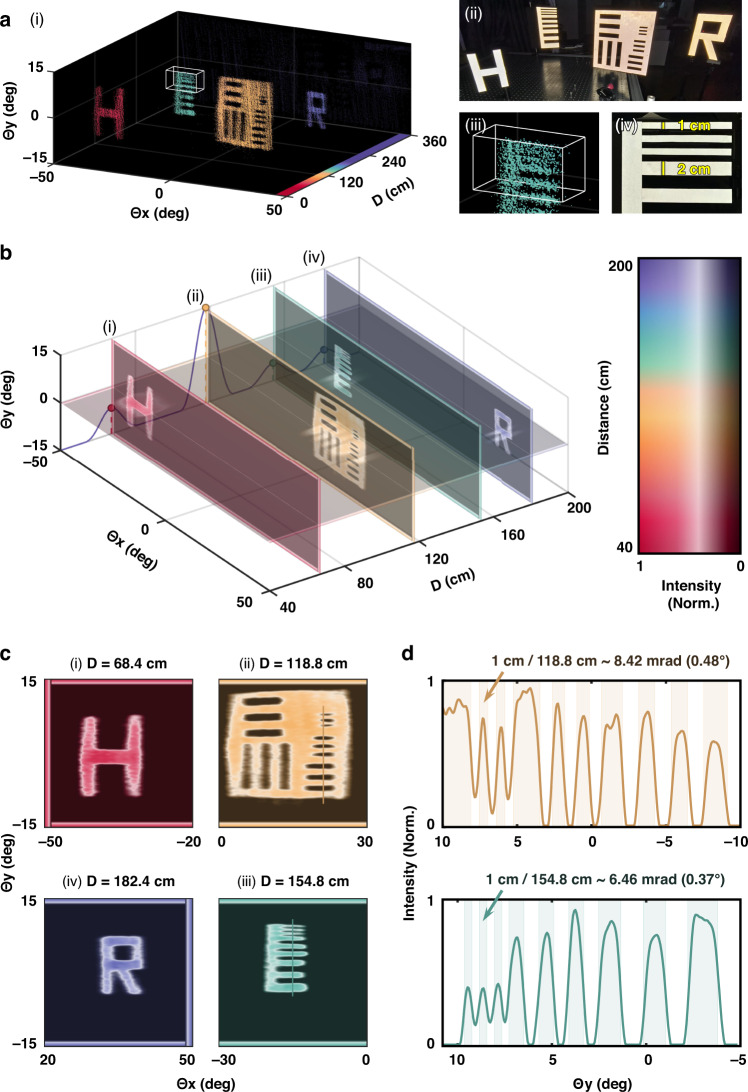
High spatial resolution 3D imaging. **a** Direct point cloud imaging results for four targets (i), alongside the corresponding physical scene (ii). Local point cloud reconstruction of a resolution target (iii) is compared with the actual object (iv). **b** Depth-resolved intensity slices recovered through subpixel reconstruction, with (i)–(iv) showing intensity distributions at four distinct distances, respectively. Distance is color-coded, while intensity is represented by saturation and grayscale. The purple line at *Θ*_*x*_ = -50° plane represents the total intensity distribution *I*(*D*) along the distance direction. The slice at *Θ*_*y*_ = 0° represents the 2D intensity distribution *I*(*Θ*_*x*_, 0°, *D*). **c** Intensity distributions within the extracted depth slices, corresponding to the extrema of *I*(*D*) in (**b**). **d** Intensity profiles at a specific *Θ*_*x*_, with the top and bottom panels corresponding to **c**(ii) and **c**(iii), respectively. The 1 cm-wide feature, previously indistinct in the raw point cloud **a**(iii), is now clearly resolved, demonstrating a spatial resolution of 6.46 mrad

Fig. 5a illustrates the direct point cloud imaging results for four test objects (i), including the letters “H” and “R” from “High Resolution”, as well as two resolution targets with a minimum feature linewidth of 1 cm, corresponding to the photograph of the physical scene (ii)&(iv). Owing to the relatively small divergence angle ( ~ 11 mrad) of the AML output beams, we are able to resolve a linewidth of 2 cm (13 mrad angular width) in the local point clouds (iii). However, resolving finer details with a linewidth of 1 cm remains challenging.

To further enhance spatial resolution, we employ a subpixel reconstruction algorithm (see Supplementary Section 7 for details) to recover a higher-resolution 3D dataset, *I*_reconstructed_ (*Θ*_*x*_, *Θ*_*y*_, *D*), which encodes relative intensity variations across the 3D spatial domain. As depicted in Fig. 5b, four distinct depth slices are extracted based on the extrema of *I* (*D*) (marked by the purple line at *Θ*_*x*_ = -50° plane).

The detailed intensity distribution of these four slices is presented in Fig. 5c, clearly revealing object features that were unresolved in the raw point cloud data. The extracted distances—68.4 cm, 118.8 cm, 154.8 cm, and 182.4 cm—closely match the measured object positions (69 cm, 119 cm, 154 cm, and 184 cm). Intensity profiles along the *Θ*_*y*_ direction at fixed *Θ*_*y*_ values are plotted in Fig. 5d, further confirming the system’s high spatial resolution. The top and bottom panels correspond to Fig. 5c(ii) and (iii), respectively, where the resolution target features become distinctly visible in the reconstructed intensity maps. In particular, the resolution target at a distance of 154.8 cm, with a 1 cm linewidth, is clearly resolved, demonstrating a lateral spatial resolution of 6.46 mrad.

## Discussion

To conclude, we have developed a novel LiDAR architecture that integrates high spatiotemporal resolution with wide-FOV 3D imaging capabilities. Through precise inter-axis rate matching in the spectral-AO scanning mechanism, we achieve a maximum FPAR up to 36.6 MHz, ensuring high temporal resolution. In addition, through the tailored design of a wide-FOV AML, we realize a spatial resolution of 6.46 mrad (0.37°) across a 102° FOV.

While increasing the number of spectral channels *N*_*λ*_ at a given *f*_rep_ could further enhance temporal resolution, the total chirp time of spectral scanning *T*_chirp_ must satisfy:

9 $${T}_{\mathrm{chirp}}=({N}_{\lambda }-1)\cdot \Delta \tau < {T}_{\mathrm{pulse}}$$

The margin created by *T*_chirp_ < *T*_pulse_ serves two critical purposes: (i) accommodating the electrical/acoustic response time required for the AOD’s synchronized scanning; (ii) tolerating minor temporal drift (e.g., from temperature fluctuations or electronic noise). This thereby imposes an upper limit on the PPAR based on the temporal interval between adjacent time-stretched sub-pulses, Δ*τ*:

10 $${\rm{PPAR}}={N}_{\lambda }{f}_{{\rm{rep}}}=\frac{{N}_{\lambda }}{{T}_{{\rm{pulse}}}} < \frac{{N}_{\lambda }}{{T}_{{\rm{chirp}}}}=\frac{1}{\varDelta \tau }$$

In our experimental implementation with *f*_rep_ = 1.22 MHz, Δ*τ* = 24.6 ns, *N*_*λ*_ = 30, *T*_chirp_ = 713.4 ns (PPAR = 36.6 MHz), the chirp duration *T*_chirp_ can be extended to ~787 ns, enabling *N*_*λ*_ = 33 and thereby elevating PPAR to 40.2 MHz.

In single-pulse direct-TOF LiDAR, the trade-off between *f*_rep_ and the ambiguity distance *d*_m_ inherently limits the PPAR. Time-frequency multiplexing decouples this constraint, enhancing the PPAR without compromising *d*_m_ at a given *f*_rep_. Instead, the sub-pulses temporal interval Δ*τ* defines the maximum detectable depth span Δ*d*_m_ = *c*·Δ*τ*/2 = 3.69 m, creating a PPAR-Δ*d*_m_ trade-off. Theoretically, by resolving 30-pulse echo signals within a single laser pulse period *T*_pulse_, we can overcome the distance limitation imposed by Δ*τ*, yielding an ambiguity distance up to *d*_m_ = *c*·*T*_pulse_/2 = 123 m (see Supplementary Section 10 for details). Additionally, applying *α*-dimensional orthogonal pulse coding combined with cross-correlation detection could further extend the ambiguity distance by *α*-fold under the same PPAR, and this approach is compatible with the time-stretching method we employed, as demonstrated in our recent work^44^.

To demonstrate the system’s capability for detecting small, fast-moving targets (required for scenarios like drone tracking), consider a racing drone with a diagonal span of ~60 cm located 50 m away and traveling at a horizontal speed of 180 km/h. The drone would traverse the entire 100° FOV in ~2.38 s. With our system operating at 183.5 fps (*β*_1_ = 4), the drone could be captured in 437 consecutive frames, enabling smooth motion tracking. Furthermore, the system’s angular resolution of 6.46 mrad, ranging precision of 3 cm, and ambiguity distance of 123 m are all sufficient for detecting such a drone. If both the *x* and *y* AODs employ pixel-by-pixel scanning, the total number of addressable points becomes 7×83×30 = 17,430, enabling a higher imaging frame rate of 2,098 fps (*β*_1_ = 1) to meet the detection requirements for faster-moving objects. Considering the Nyquist limit, which necessitates capturing 4 frames to recover the object’s speed, our system is theoretically capable of detecting objects moving at speeds of up to 225 mega-meter/h at 50 m away.

Moreover, in spectral scanning, the dispersion capability is constrained by the grating’s line density. Employing a grating with a higher line density could further enhance the horizontal FOV. Additionally, 2D spectral scanning could be achieved by integrating a diffraction grating with a virtually imaged phased array (VIPA)^18,45^, enabling more efficient utilization of the spectrum and the scanning space.

Regarding modulation bandwidth, the currently used arrayed waveguide grating (AWG) for wavelength division multiplexing exhibits a 3 dB bandwidth of 0.28 nm. However, the narrow 0.4 nm channel spacing causes spectral overlap and crosstalk, causing overlapping spots in the grating’s dispersion direction and degrading spatial resolution. In our experiment, a programmable spectral shaping device, the Waveshaper (WS), is employed to refine the comb-like output spectrum, narrowing the linewidth from 0.28 nm to ~0.1 nm, significantly improving the spatial resolution in the dispersion direction (see Supplementary Section 2). Therefore, replacing our laser source with an optical frequency comb featuring a narrower linewidth and a better-matched spectral profile could further enhance the spectral and spatial resolution.

In summary, by leveraging wide-FOV AML-coordinated spectral-AO scanning, we have successfully developed a LiDAR architecture that attains an outstanding 36.6 MHz FPAR, a precise 0.37° resolution across a 102° FOV, thus achieving high spatiotemporal resolution. Additionally, the wide-FOV and low-divergence beam characteristics enabled by planar optical components infuse renewed vitality into traditional bulk LiDAR systems. This technology holds promise for applications in non-line-of-sight imaging^46,47^, inverse synthetic aperture LiDAR, and other imaging systems requiring scanning modules. To achieve longer-range detection, frequency up-conversion^48^ on echo signals can be employed to convert near-infrared light into the visible spectrum, aligning with the high responsivity range of mature silicon-based photodetectors and thereby improving detection efficiency. In the future, it is worth exploring whether leveraging the orbital angular momentum (OAM) modes for orthogonal spin multiplexing, in conjunction with composite phase metasurfaces for spin demultiplexing, can facilitate parallel beam scanning. Recently, parallel detection methods for high-speed LiDAR have also emerged, including multi-detector architectures^7,21,49^, optical spectro-temporal coding^20^, parallel chaos^22^, and code-division multiple access (CDMA) methods^44,50^; these could further enhance the LiDAR’s temporal resolution. Furthermore, our single-transceiver architecture can also be extended to parallel detection and holds promise for application in integrated sensing and communication systems, enabling faster and more precise spatiotemporal perception.

## Materials and methods

### Experimental methodology

The supercontinuum laser (NKT Photonics, SuperK EXTREME EXR-15) generates broad-spectrum pulsed light, which is then amplified by an erbium-doped fiber amplifier (EDFA) with a gain spectrum centered around 1550 nm. Subsequently, time-frequency multiplexing produces 30 comb-like spectral channels, each featuring a linewidth of 0.28 nm and a spacing of 0.4 nm (details in Supplementary Section 2). These channels are further refined to a linewidth of 0.1 nm using a Waveshaper (Coherent, Waveshaper 4000 A). The collimated output beam is directed through a dual-axis AOD (AA Opto-electronic, DTSXY-A6-1550), enabling 2D acousto-optic scanning of the [1, 1] diffraction order, which is filtered by a pinhole aperture. The AODs are driven by a radio frequency (RF) generator (AA Opto-electronic, DDSPA-B415b-0) with external amplifiers, and the driving module is synchronized with the laser pulses via a field programmable gate array (FPGA) to perform precise rate matching. The dual-axis AOD outputs a vertically polarized beam, which is rotated to horizontal polarization by passing through an HWP oriented at 45° to enhance responsiveness to the vertical blazed grating (LBTEK, BG25-600-1500) with the blaze angle of 26.78°. After dispersion by the grating for spectral-AO scanning, the beams are directed onto the AML through a focusing lens (LBTEK, MBCX10611), enabling an expanded FOV. The AML was fabricated by electron beam lithography techniques with the fabrication flow chart shown in Supplementary Fig. S4d. The target’s echo signals first pass through a bandpass filter (BPF) (THORLABS, FBH1550-40) to suppress most of the ambient stray light by narrowband spectral filtering. They are then detected by a PMT (HAMAMATSU, H10330C-75) and digitized by a data acquisition card (Teledyne, ADQ7DC) operating at a sampling rate of 5 GS/s. Data post-processing, including amplitude filtering, frequency-domain filtering, three-point fitting, point cloud denoising, and sub-pixel reconstruction, is performed to refine the 3D morphology of the objects (details in Supplementary Section 7).

## Supplementary information

Supplementary information

Dynamic 3D imaging of a high-speed rotating fan in the xy-plane

Dynamic 3D imaging of two rotating cylindrical targets in the xz-plane

Dynamic 3D imaging of a 3 kHz chopper

## Data Availability

All data needed to evaluate the conclusions in the paper are present in the paper and/or the Supplementary Materials. Additional data related to this paper may be requested from the authors.
