# Supplementary material for: Spectral-acoustic-coordinated astigmatic metalens for wide field-of-view and high spatiotemporal resolution 3D imaging
Source: Light Sci Appl. 2026 Jan 23;15:85. doi: 10.1038/s41377-025-02180-7 (PMC12830631; doi:10.1038/s41377-025-02180-7)
Supplement: Supplementary file 1 — Supplementary information [file 41377_2025_2180_MOESM1_ESM.pdf]

Supplementary Information for

Spectral-acoustic-coordinated astigmatic metalens for wide field-of-view and high spatiotemporal resolution 3D imaging

Shujian Gong[1,2,3†], Yinghui Guo[1,2,3,4†], Xiaoyin Li[1,2†], Mingbo Pu[1,2,3,4*], Peng Tian[1,2], Qi Zhang[1,2], Lianwei Chen[1,2], Wenyi Ye[1,2,3], Heping Liu[5], Fei Zhang[1,2], Mingfeng Xu[1,2], and Xiangang Luo[1,2,3*]

[1] State Key Laboratory of Optical Field Manipulation Science and Technology, Institute of Optics and Electronics, Chinese Academy of Sciences, Chengdu 610209, China.

[2] Research Center on Vector Optical Fields, Institute of Optics and Electronics, Chinese Academy of Sciences, Chengdu 610209, China.

[3] College of Materials Sciences and Opto-Electronic Technology, University of Chinese Academy of Sciences, Beijing 100049, China.

[4] Sichuan Provincial Engineering Research Center of Digital Materials, Chengdu, 610213, China.

[5] Tianfu Xinglong Lake Laboratory, Chengdu, 610213, China.

†These authors contributed equally to this work.

*Email: pmb@ioe.ac.cn, lxg@ioe.ac.cn

This PDF file includes:

Section 1: Comparisons among related single-channel LiDARs.
Section 2: Low-crosstalk time-frequency multiplexing enabled by programmable spectral shaping.
Section 3: Spectral-acousto-optic scanning cascading the AML.
Section 4: Optimization design and fabrication of the AML.
Section 5: Characterization of the beam deflection angle and FOV.
Section 6: Characterization of the beam divergence angle.
Section 7: Data processing procedure.
Section 8: Discussion on ranging resolution.
Section 9: Power loss analysis & discussion on long-range detection.
Section 10: Maximum detectable depth span (Δd_m) and ambiguity distance (d_m).

Other supplementary materials for this manuscript include the following:

Movie S1: Dynamic 3D imaging of a high-speed rotating fan in the *xy*-plane.
(Parameters: AOD scan points: 20 × 83; full-field scan points: 20 × 83 × 30; frame rate: 183.5 fps at $\beta = 4$, 367 fps at $\beta = 2$, and 734 fps at $\beta = 1$)

Movie S2: Dynamic 3D imaging of two rotating cylindrical targets in the *xz*-plane.
(Parameters: AOD scan points: 20 × 83; full-field scan points: 20 × 83 × 30; frame rate: 122.3 fps at $\beta = 6$)

Movie S3: Dynamic 3D imaging of a 3 kHz chopper.
(Parameters: AOD scan points: 1 × 60; full-field scan points: 1 × 60 × 30; frame rate: 20.3 × 10^3 fps at $\beta = 1$ and 10.2 × 10^3 fps at $\beta = 2$)

Supplementary Section 1: Comparisons among related single-channel LiDARs.

Table S1 | Comparison of the performance metrics of similar single-channel transceiver LiDARs.

Ref.	Scanning mechanism	PPAR (MHz)	β (fast axis line rate / slow axis point rate)	FPAR (MHz)	Addressable points (@ frame rate)	Fast axis FOV	Fast axis angular resolution θ	$C_{spatial\text{-}1D}$ (rad) [b)]
**This work**	Spectral scanning + 2-axis AOD	36.56	1	36.56	600 × 83 @ 734 fps	102°	~0.37°	487.6
**Ref.**[1]	Spectral scanning + mechanical scanning	30	50 [a)]	~0.6	30 × --	7°	~0.23°	3.67
		88	17 [a)]	~5.12	256 × --	9°	~0.04°	40.2
**Ref.**[2]	2-axis AOD	6.25	1	6.25	83 × 83	150°	~1.8°	217.3
**Ref.**[3]	Spectral scanning + mechanical scanning	21.38	24 [a)]	~0.9	45 × 33	2°	~0.044°	1.57
**Ref.**[4]	Spectral scanning + mechanical scanning	7.6	1	7.6	475 × 1000 @ 16 fps	7.1°	~0.015°	58.9
**Ref.**[5]	Spectral scanning + mechanical scanning	4.1	96	~0.04	51 × 75	2°	~0.04°	1.78
**Ref.**[6]	Spectral scanning + mechanical scanning	5.6	1000	~0.0056	28 × 136	~2°	~0.06°	1.95

a) If not mentioned in the text, the mechanical scanning is calculated based on a maximum point-switching rate of 20 kHz.

b) Calculated by the fast axis ($C_{spatial\text{-}1D} = N{\cdot}FOV = FOV^2/\theta$).

Table S1 and Fig. S1 present a comparison of the performance metrics of similar single-channel transceiver LiDAR systems. These systems typically face one or more limitations: some suffer from a narrow FOV (Refs.[1, 3, 4, 5, 6]), some experience inter-axis rate mismatch that results in frame-wise point acquisition rate (FPAR) dropping below pixel-wise point acquisition rate (PPAR) (Refs.[1, 3, 4, 5, 6]), and some encounter beam divergence that reduces the number of resolvable points (Ref.[2]). Taken together, these issues lead to suboptimal comprehensive spatiotemporal detection capabilities.

In contrast, in this work, we first improve the FPAR through rate matching enabled by spectral-acousto-optic (spectral-AO) scanning. Then, to address the FOV mismatch in heterogeneous dual-axis cascade scanning, we uniquely design an astigmatic metalens (AML) to correct beam astigmatism and field distortion caused by spectral-AO scanning, while simultaneously expanding the FOV. This approach ultimately enables exceptional comprehensive spatiotemporal detection capability.

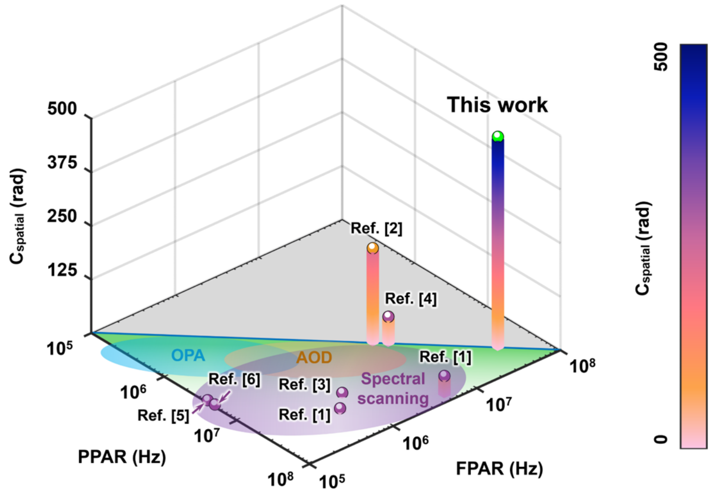

Fig. S1 | Comparison of this work with related studies. The three coordinate axes represent PPAR, FPAR, and spatial detection capability ($C_{spatial\text{-}1D}$). This work achieves both high FPAR and $C_{spatial\text{-}1D}$, demonstrating superior spatiotemporal detection capabilities.

Supplementary Section 2: Low-crosstalk time-frequency multiplexing enabled by programmable spectral shaping.

Figure S2 provides a detailed illustration of how the spectro-temporal encoding module achieves time-frequency multiplexing through discrete time-stretching. Fig. S2a depicts the specific experimental setup, which mainly consists of a pair of arrayed waveguide gratings (AWGs), 30 variable optical attenuators (VOAs) for balancing the output power, and 30 single-mode fibers for time-stretching. AWG1 demultiplexes (DeMUX) the input broadband laser source, producing 30 spectral channels with equal wavelength spacing. In an ideal scenario, these 30 channels transmit through fibers of lengths in an arithmetic progression and are subsequently combined by AWG2, functioning as a wavelength division multiplexer (MUX). This results in the output of discrete chirped pulse sequences with perfectly equal temporal intervals ($\Delta\tau$ = 24.6 ns) and equal wavelength spacing ($\Delta\lambda$). Based on this, we can calculate the required common difference in fiber length (ΔL):

$$\Delta L = \frac{c}{n_{\text{eff}}} \cdot \Delta\tau \approx 5.03 \text{ m} \tag{S1}$$

where c denotes the speed of light in vacuum and n_{eff} represents the effective refractive index of the fiber core. In actual operation, by increasing the common difference in the delay-fiber length of adjacent channels, we can correspondingly increase the time-separation $\Delta\tau$ of the 30 spectral channels, thereby increasing the non-ambiguous depth span Δd_{m}.

To ensure accurate time-of-flight (TOF) calculations, we calibrate the delay times of the 30 time-stretched sub-pulses by detecting the encoding module's output signal, using the laser pulse as a timing reference, as shown in Fig. S2b. The horizontal axis represents the channel number, where channel No. 1 (Ch #1) corresponds to λ_1 = 1541.7 nm and channel No. 30 (Ch #30) corresponds to λ_{30} = 1553.3 nm. Fig. S2b(i) shows that the delay times across channels increase almost linearly, consistent with expectations. Fig. S2b(ii) displays the pulse intervals between adjacent channels. Due to deviations in fiber length differences (ΔL) from preset values, the intervals are non-uniform, with the minimum value (~23.4 ns) slightly lower than the average (~24.6 ns).

In our experiment, the laser source (NKT Photonics, SuperK EXTREME EXR-15) generates a supercontinuum spectrum spanning 500-2000 nm, which more than adequately covers the operational wavelength range of 1541-1554 nm, as illustrated in Fig. S2c(i). The total output spectrum after time-stretching is presented in Fig. S2c(ii). The AWG splits the original signal into 30 channels with a wavelength interval of $\Delta\lambda$ = 0.4 nm. Although these channels are temporally separated through time-stretching, each channel exhibits a 3 dB bandwidth of 0.28 nm, leading to some spectral overlap and crosstalk between adjacent channels (as shown in the inset of Fig. S2c(ii)). This spectral overlap degrades the spectral resolution and, consequently, reduces the spatial resolution along the horizontal direction after grating dispersion.

To address this issue, we introduced a programmable spectral shaping device known as a Waveshaper (WS), a passive optical component capable of arbitrarily modifying the spectral profile of incident light. In our setup, the WS (Coherent, Waveshaper 4000A) was configured to perform spectral-domain filtering in a comb-like pattern, with each comb tooth centered at the wavelength corresponding to the 30 channels of the AWG, and a linewidth of 0.13 nm. This configuration allows each channel from the AWG to undergo narrowband filtering through the WS, while also enabling uniform channel intensities via differential spectral attenuation. Fig. S2c(iii) illustrates the spectrum after time-stretching and subsequent WS filtering. Compared to the AWG output spectrum shown in Fig. S2c(ii), the channels exhibit narrower linewidths with reduced crosstalk, thereby significantly enhancing the spatial resolution in the grating's dispersion direction.

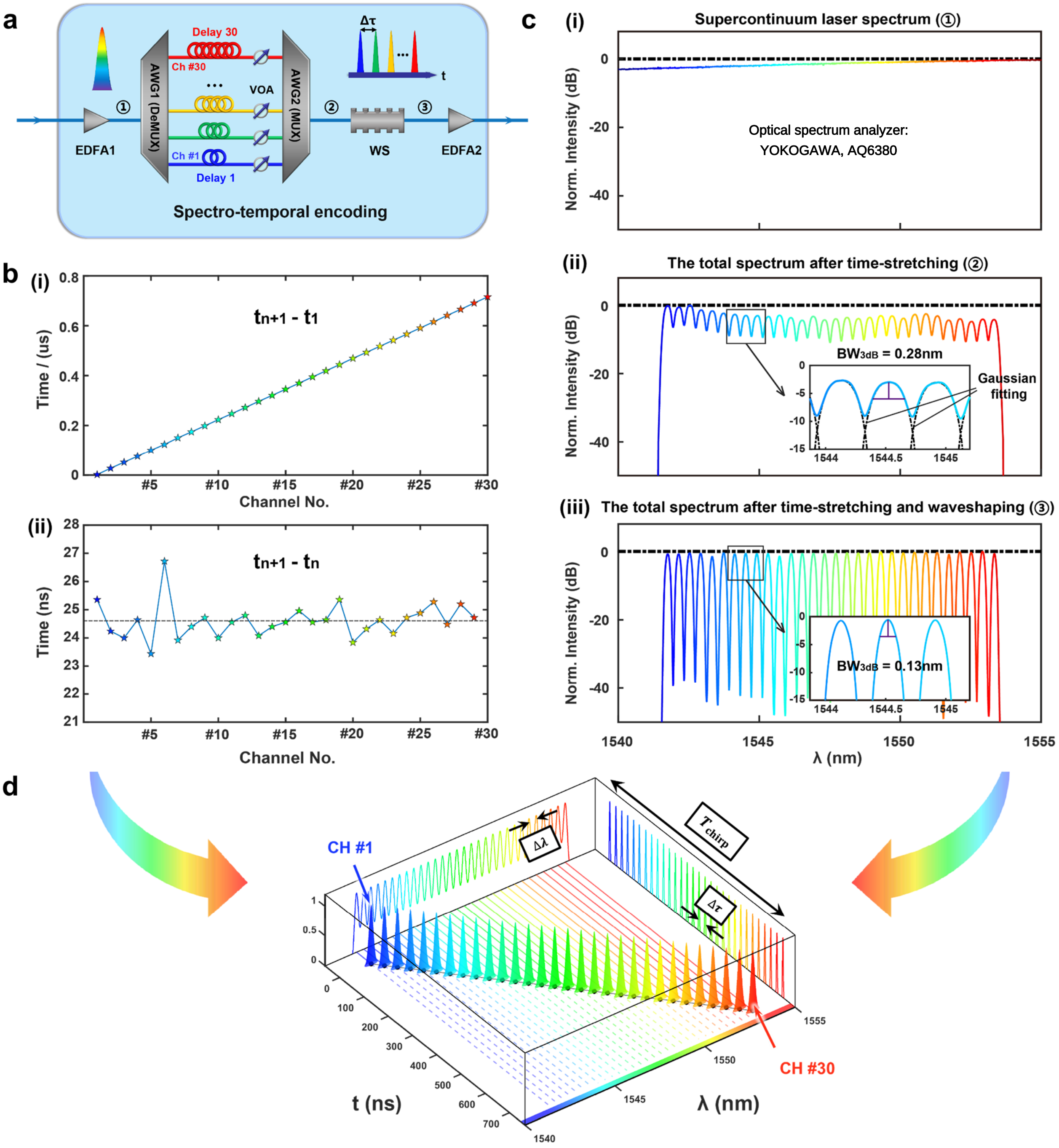

Fig. S2 | Implementation of time-frequency multiplexing. (**a**) Spectro-temporal encoding module. It primarily consists of two arrayed waveguide gratings (AWGs) and several fibers. The AWGs split the source into 30 channels with equal spectral intervals and then combine them. Each channel propagates through fibers of increasing lengths, resulting in time-stretched time-frequency mapping depicted by (**d**). EDFA: erbium-doped fiber amplifier; VOA: variable optical attenuator; WS: waveshaper. (**b**) (i) Relative time delay of the emitted pulses compared to the first channel (Ch #1); (ii) Relative time delay between emitted pulses of adjacent channels. (**c**) Spectral variations at each stage of time-frequency multiplexing: (i) Spectrum of the incident laser source; (ii) Total spectrum of the 30 channels after time-stretching. The inset shows spectral overlap between adjacent channels; (iii) Total spectrum after time-stretching and waveshaping. The inset shows significant suppression of channel crosstalk. (**d**) The achieved time-frequency mapping features an equal time interval of $\Delta\tau = 24.6$ ns, an equal wavelength interval of $\Delta\lambda = 0.4$ nm between adjacent channels, and a total chirped pulse sequence duration of $T_{chirp} = 713.4$ ns.

Supplementary Section 3: Spectral-acousto-optic scanning cascading the AML.

3.1 Spectral-dual-AO scanning cascading the AML for wide-FOV high spatiotemporal resolution 3D imaging.

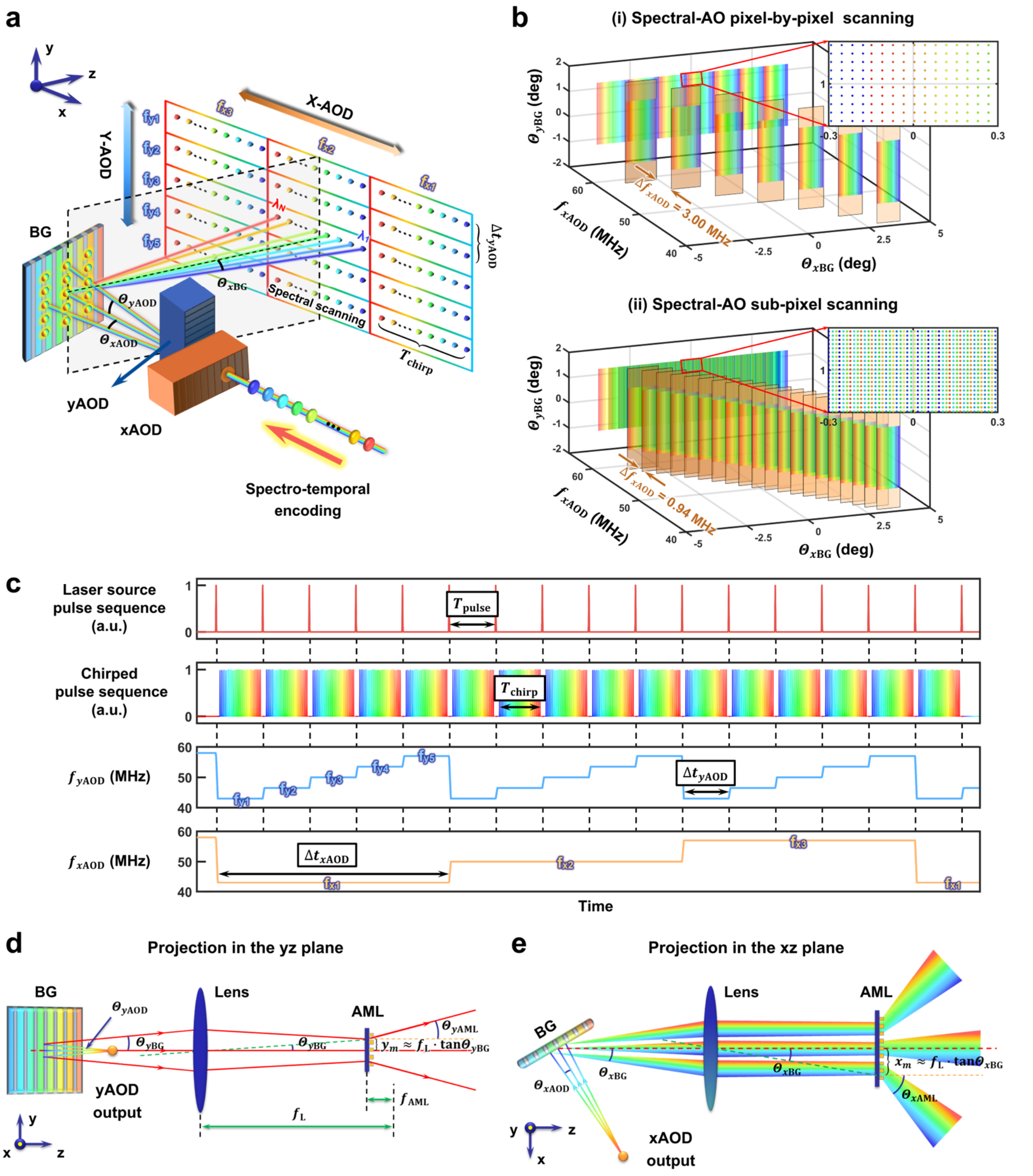

Fig. S3 | Spectral-acousto-optic scanning cascading the AML. (**a**) Schematic of the spectral dual-AO cascade scanning. (**b**) Implementation of (i) pixel-by-pixel scanning and (ii) sub-pixel scanning in spectral-AO scanning. (**c**) Timing diagrams of spectral-dual-AO scanning corresponding to **a**. As an example, consider a complete scan frame comprising 5 *y*AOD and 3 *x*AOD scanning positions. After completing a spectral scan, the *y*AOD swiftly transitions to the next position to achieve rate matching (within the time gap T_{pulse}-T_{chirp}), while the *x*AOD operates in a similar manner. We achieve this synchronization via a field-programmable gate array (FPGA). Meanwhile, to ensure reliable synchronized scanning, the sub-pulses generated by time-stretching must fully reside within the intervals between AOD frequency-switching events; the alignment of these sub-pulses with the AOD's stable regions is realized using a single-mode fiber of a specific length to introduce a required time delay. (**d**)(**e**) Schematic of the AML extending the FOV in two orthogonal directions. (**d**) In the *yz*-plane, the BG acts as a planar mirror, with the vertical FOV extending solely through the AML. (**e**) In the *xz*-plane, the BG introduces spatial dispersion that extends the initial horizontal FOV, which is further magnified by the combination of the lens and AML, functioning similarly to a Galilean telescope.

The schematic diagram of the spectral-dual-AO cascade scanning is illustrated in Fig. S3a, with the corresponding scanning timing diagram presented in Fig. S3c. Since the vertically oriented grating only provides spatial dispersion in the horizontal direction, the beam propagation in the two directions will not be symmetric.

In the vertical plane (*yz* plane) depicted by Fig. S3d, the scanning direction of *y*AOD is parallel to the orientation of the blazed grating (BG), causing the grating to merely function as a reflective mirror. Here, the scanning angles of the dual-axis AOD (AA Opto-electronic, DTSXY-A6-1550) are governed by the acousto-optic (AO) Bragg diffraction mechanism[7]. Specifically, tuning the *y*AOD's driving frequency ($f_{y\mathrm{AOD}}$) from 41 to 59 MHz yields an output angle range of $\Theta_{y\mathrm{AOD}} \in [-1.2°, 1.2°]$, which in turn results in BG's output angle of $\Theta_{y\mathrm{BG}} = \Theta_{y\mathrm{AOD}} \in [-1.2°, 1.2°]$.

In the horizontal plane (*xz* plane) depicted by Fig. S3e, the scanning direction of *x*AOD is orthogonal to the BG's orientation, resulting in spatial dispersion with an angle extension of $\Theta_{x\mathrm{BG}}$, calculated by the grating equation:

$$\Theta_o = \arcsin\left(\frac{\lambda_n}{d} - \sin\Theta_{i\_x(n)}\right) \tag{S2}$$

$$\Theta_{x BG} = \Theta_o(\lambda_n, \Theta_{i\_x(n)}) - \overline{\Theta_o} \tag{S3}$$

where λ_n is the wavelength of the incident light, n = 1, 2, 3, …, 30 denotes the channel number, and d is the grating constant (1/600 mm). The angle $\Theta_{i\_x(n)}$ represents the incident angle at the grating and also corresponds to the *x*AOD's output angle at its $x(n)$-th scanning position, while Θ_o is the diffracted angle of the grating. Eq. S3 is used to reference the angle 0° because the optical axis also shifts upon diffraction. Moreover, Eq. S2 reveals a nonlinear relationship between the output angle and both the incident angle and wavelength, leading to the slight asymmetry of the horizontal output angle of the BG, as shown in Fig. S3b(i).

Based on Eq. S3, we can determine the output angle for *x*AOD and the corresponding driving frequencies $f_{x\mathrm{AOD}}$ to ensure that adjacent spectral scanning fields in the horizontal direction connect seamlessly without gaps, expressed as:

$$\Theta_o(\lambda_{30}, \Theta_{i\_x(n)}) \approx \Theta_o(\lambda_1, \Theta_{i\_x(n+1)}) \tag{S4}$$

From Eq. S4, 7 scanning positions for *x*AOD are derived, with $f_{x\mathrm{AOD}}$ = 41, 44, 47, …, 59 MHz, yielding an output angle range of $\Theta_{x\mathrm{AOD}} \in [-1.2°, 1.2°]$, as illustrated in Fig. S3b(i). Here, *y*AOD scans 83 positions; combined with 30 spectral channels, this yields a total of 7×83×30 scanning positions, corresponding to the scanning mode in Fig. **3**c. Further calculations using Eqs. S2 and S3 yield the horizontal output angle range of the BG: $\Theta_{x\mathrm{BG}} \in [-4.0°, 4.0°]$. Additionally, we perform finer scanning for higher resolution, resulting in a denser subpixel scanning lattice of 20×165×30 points (20 points for *x*AOD and 165 for *y*AOD; as shown in Fig. S3b(ii), corresponding to the scanning mode in Fig. **5**). Furthermore, for the experiments in Fig. **4**a–d, the *x*/*y*AOD scan 20 and 83 points, respectively.

Consequently, due to horizontal dispersion, the BG's output forms a rectangular FOV ($\Theta_{x\mathrm{BG}} \in [-4.0°, 4.0°]$ *vs.* $\Theta_{y\mathrm{BG}} \in [-1.2°, 1.2°]$; FOV: ~8.0° × 2.4°). Thus, the designed wide-FOV metalens also requires a phase profile over a rectangular area to match the scanning spots formed after focusing by the front lens.

The angular magnification of such a system resembling a Galileo telescope is given by $M = |f_\mathrm{L} / f_\mathrm{AML}|$. Since the effective focal length f_AML of the AML is limited by the periodicity of the meta-atoms, a longer focal length f_L for the focusing lens is preferable. However, with the maximum size of the AML set at r_m = 5 mm, it is crucial to ensure that the focused light spot falls within its effective area while leaving some margin:

$$\sqrt{x_\mathrm{m}^2 + y_\mathrm{m}^2} \approx f_\mathrm{L} \cdot \sqrt{\tan^2\Theta_{x\mathrm{BG\,max}} + \tan^2\Theta_{y\mathrm{BG\,max}}} < 0.9\ r_\mathrm{m} \tag{S5}$$

where x_m and y_m represent the coordinates of the edges of the rectangular scanning area. This leads to the condition f_L < 62 mm, prompting us to select f_L = 60 mm (LBTEK, MBCX10611) to maximize the angular magnification. Next, we optimize the phase distribution of the AML using commercial software (*Zemax OpticStudio*) to achieve a larger horizontal FOV while effectively suppressing beam divergence, as detailed in Supplementary Section 4. The optimized phase profile, expressed by Eq. (**8**) in the main text, is shown in Fig. **3**b, with the corresponding phase coefficients listed in Table S2.

Table S2 | Phase coefficients of the AML.

c_1	c_2	c_3	c_4	c_5	c_6	c_7	c_8	c_9
10070	8099.4	-712.23	403.15	1200.0	260.88	320.00	320.00	320.00

The above coefficients represent the asymmetric modulation in the x and y directions, which reflect the astigmatic characteristics of the AML. By following the standard form of the non-astigmatic defocus quadratic phase[8] $\Phi(r) = k_0 r^2/2f$, where $k_0 = 2\pi/\lambda$, the effective focal lengths of the AML in the x/y direction, $f_{\mathrm{AML}\_x,y}$, can be approximately derived as:

$$f_{\mathrm{AML}\_x} \approx \frac{\pi r_m^2}{c_1 \lambda} = 5.0\ \mathrm{mm}\ ,\ f_{\mathrm{AML}\_y} \approx \frac{\pi r_m^2}{c_2 \lambda} = 6.2\ \mathrm{mm} \tag{S6}$$

Based on their ratios with f_{L}, the angular magnification M is ~12-fold/9.7-fold in the x/y direction relative to the BG's output angles. These values agree closely with the simulated output angles $\Theta_{x\mathrm{AML}} \in [-51°, 51°]$ and $\Theta_{y\mathrm{AML}} \in [-13°, 13°]$ (see Fig. **3**c in the main text; FOV: 102° × 26°). Minor discrepancies between theory and simulation arise primarily from higher-order phase modulation effects at the FOV edges, where non-quadratic terms (e.g., quartic, sixth-order) refine the wavefront beyond simple defocus-like behavior.

3.2 Spectral-AO scanning for ultra-high-speed 3D imaging.

The demonstration experiment in Fig. **4**e aims to verify the ultra-high-speed dynamic 3D imaging capability of our all-solid-state spectral-AO scanning mechanism—which achieves an FPAR of up to 36.6 MHz and supports an ultra-high frame rate of up to 20.3 kfps for 60×30 pixels per frame. As a validation of the system's high-speed performance (serving as a supplement to the wide-FOV, high spatiotemporal resolution LiDAR in the main workflow), the scanning FOV in this mode is intentionally limited to prioritize high-speed performance.

Specifically, the xAOD's driving frequency is fixed at 50 MHz (AOD's central frequency) such that the beam from the yAOD scans in the vertical plane perpendicular to the grating, leading to a grating incidence angle of $\Theta_i = 0°$. Thirty spectral channels enable horizontal beam scanning via grating diffraction; thus, the horizontal FOV of ~1.1° is determined by the grating equation (Eq. **S2**): $\Delta\Theta_o = \arcsin(\lambda_{30}/d) - \arcsin(\lambda_1/d)$. In contrast, the yAOD scans 60 points along the vertical direction, with a total frequency sweep bandwidth of 14.64 MHz—this yields a total vertical FOV of 2.0°, as derived from the Bragg diffraction mechanism.

Supplementary Section 4: Optimization design and fabrication of the AML.

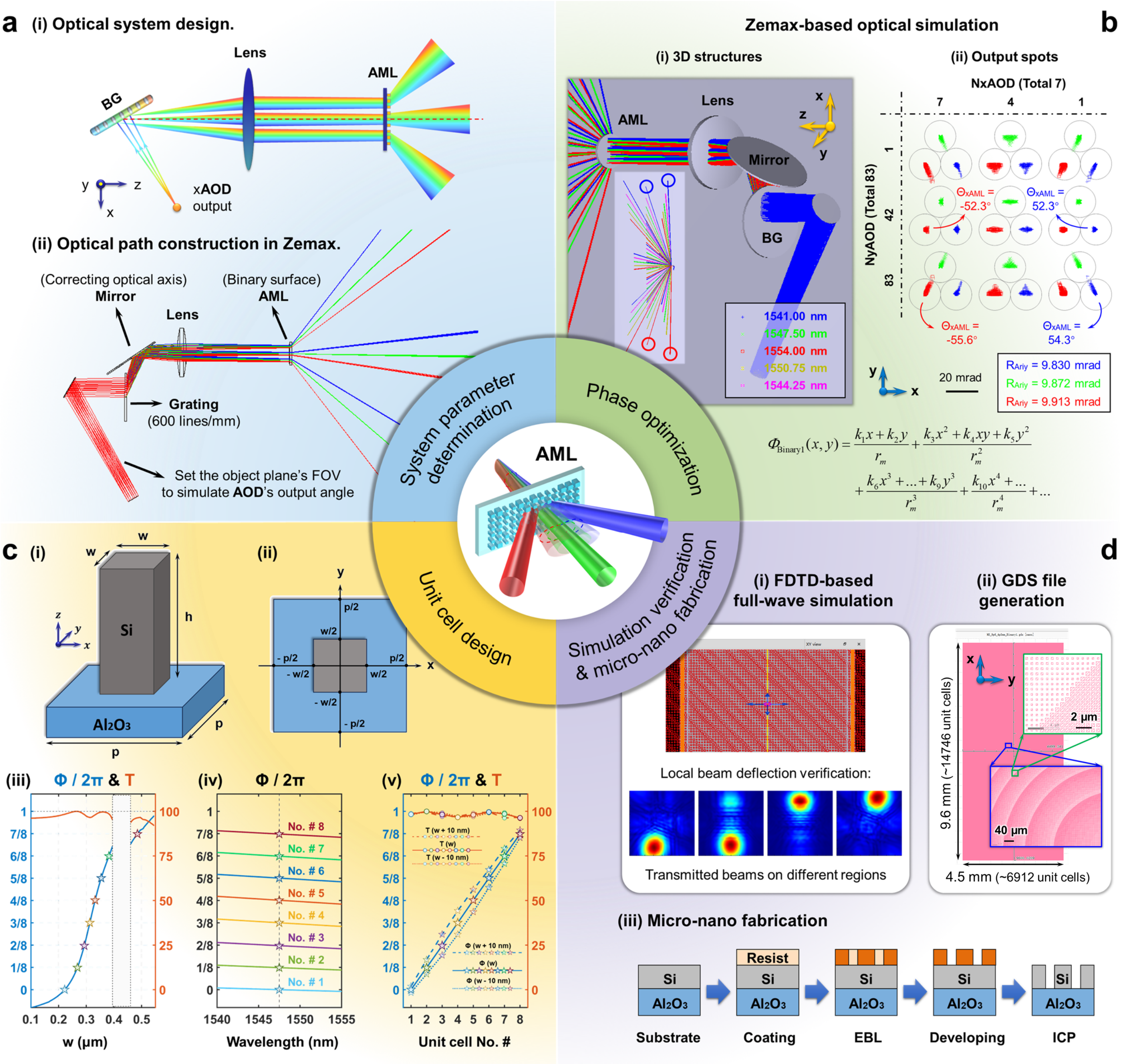

$$\Phi_{\text{Binary1}}(x,y)=\frac{k_1x+k_2y}{r_m}+\frac{k_3x^2+k_4xy+k_5y^2}{r_m^2}+\frac{k_6x^3+\ldots+k_9y^3}{r_m^3}+\frac{k_{10}x^4+\ldots}{r_m^4}+\ldots$$

Fig. S4 | Optimization design and fabrication of the AML. (**a**) System parameter determination. (i) Optical system design, as detailed in Fig. S3. (ii) Optical path construction in *Zemax* is based on the component parameters determined in the previous step. (**b**) Phase optimization. (i) 3D structures in *Zemax*. The inset shows the spatial distribution of the AML's output beams. (ii) Spot diagram distributions at different AOD scanning positions: The four indicated angles correspond to the four positions marked in (i). The circular boundary in each subplot denotes the Airy disk radius. (**c**) Unit cell design. (i) 3D and (ii) top-view schematics of the unit cell. The nanopillars and substrate are composed of silicon (Si) and sapphire (Al_2O_3), respectively. The nanopillars are placed at the center of a square unit cell. Fixed structural parameters: height h = 800 nm and lattice constant p = 651 nm. (iii) Parameter scanning curves of nanopillars' width w at the center wavelength of 1547.5 nm. (iv) Simulated phase responses of selected 8 unit cells across the operational wavelength range of 1540-1555 nm. (v) Simulated phases and transmissivities of the 8 unit cells at 1547.5 nm, with dashed lines illustrating the phases and transmissivities when there is a ±10 nm fabrication error in the nanopillars' width w. (**d**) Simulation verification & micro-nano fabrication. (i) FDTD-based full-wave simulation is conducted to verify local beam deflection on different regions of the AML. (ii) GDS file generation. The unit cell structures after 8-level phase quantization are used to generate the GDS file for the entire AML. This file corresponds to an AML with an overall size of 9.6 mm × 4.5 mm and contains approximately 14,746 × 6,912 unit cells, all arranged in a square lattice pattern, as shown in the inset. (iii) The micro-nano fabrication flow chart of the AML.

4.1 Core optimization objectives

The fundamental design goal of the AML in this work is to tackle the universal challenge in LiDAR systems—the inherent trade-off between a wide FOV and high spatial resolution. While a quadratic phase-based wide-FOV metalens[8] has achieved an ultra-wide 178° FOV in passive imaging[9], it has not been applied to active laser imaging with single-pixel detection. Specifically, we aim to resolve key bottlenecks of the proposed spectral-AO dual-axis scanning system: the narrow FOV (8.0° × 2.4°), beam astigmatism (elliptical beam induced by grating anisotropy), and FOV distortion. Against this backdrop, the core optimization objectives of the AML are clearly defined as follows: (i) FOV expansion: Expand the incident small-FOV beam to over 100° × 25°, meeting the FOV requirements of common commercial automotive LiDARs. (ii) Astigmatism correction: Suppress the divergence angle difference between the grating's dispersion direction (horizontal) and reflection direction (vertical), restoring the beam cross-section from an "elliptical" to a "near-circular" profile to ensure spatial resolution.

4.2 Optimization design flow

The AML design follows a logic of "system requirements → optical simulation → structural implementation → functional verification" and integrates tools such as *Zemax*, *FDTD*, and *Matlab* to achieve full-process optimization. The specific steps are as follows:

(1) System parameter determination

Based on the optical system design detailed in Supplementary Section **3**, key system parameters—including scanning angle parameters and specific model parameters of each optical component—were obtained. These parameters were used to construct the optical path in *Zemax*, as illustrated in Fig. **S4**a. The optical path was established as "AOD output → grating → mirror → lens → AML → output beams", with the following modeling details in *Zemax*:

(i) Component parameter setting: The object-space FOV was set according to the AOD output angle. The grating density was set to 600 lines/mm (a transmissive grating was used in *Zemax* for simulation; while a reflective blazed grating was employed in practice, their dispersion characteristics are determined by the grating constant, enabling equivalent simulation). The mirror behind the grating was angle-adjusted to correct the optical axis direction after grating diffraction. The focal length of the lens was set to f_L = 60 mm (as specified in Supplementary Section **3**).

(ii) Wavelength coverage: Five wavelengths were loaded simultaneously—1541.00 nm (shortest), 1544.25 nm, 1547.50 nm, 1550.75 nm, and 1554.00 nm (longest)—to ensure optimization effectiveness across the wide spectrum.

(iii) AML phase configuration: Drawing inspiration from the quadratic phase of wide-FOV metalenses[8, 9, 10], the AML was defined as a "Binary Surface", with the *Binary1* surface type (non-radially symmetric phase profile) selected instead of *Binary2* (radially symmetric). *Binary1* provides independent astigmatic phase modulation degrees of freedom in the x- and y-directions, satisfying both astigmatism correction and asymmetric FOV expansion requirements. Furthermore, odd-order terms (e.g., x, y, xy, x^3, x^2y) in the *Binary1* phase distribution were removed while even-order terms (including quadratic, quartic, and sixth-order terms) were retained to ensure the phase exhibits axial and central symmetry, as shown in Eq. (**8**) of the main text. Here, r_m = 5 mm is the normalized radius of the AML's effective region, and the phase coefficients c_i (i = 1, 2, …, 9) are the variables to be optimized.

(2) Phase optimization

(i) Merit functions definition: We defined multiple merit functions to achieve comprehensive optimization of FOV, divergence angle, and distortion. The *READ* function was used to evaluate the output FOV angle; The *RSCH* and *RSRH* functions were used to assess the beam divergence angle; The *CENX*, *RAGB*, and *DIFF* functions were used to evaluate FOV distortion; The *XDLT* and *XDGT* functions were used to limit the phase coefficients of the *Binary1* Surface (preventing exceedance of the maximum phase gradient achievable by the metalens); The *REAR* and *OPLT* functions were used to ensure the incident beams on the Binary Surface remain within the AML's effective region.

(ii) Acquisition of phase coefficients: To determine the optimal order of terms to retain in the *Binary1* phase

distribution, we compared five configurations: Second-order non-astigmatic phase, Second-order astigmatic phase, Fourth-order astigmatic phase, Sixth-order astigmatic phase (finally selected), and Eighth-order astigmatic phase. For each configuration, *Zemax*'s built-in "damped least squares method" was employed to iteratively optimize the previously defined merit functions, aiming to minimize their weighted sum by adjusting both the phase coefficients of the *Binary1* surface (quadratic, quartic, sixth-order, and eighth-order terms) and key system parameters (e.g., component distances). After optimization convergence, the final phase coefficients for each of the five configurations were obtained, each balancing FOV coverage, spot size, and distortion to the best extent possible. These results are summarized in Table S3.

Table S3 | Comparison of optimized optical performance across different phase orders.

Phase coefficients	Terms	Second-order non-astigmatic phase	Second-order astigmatic phase	Fourth-order astigmatic phase	Sixth-order astigmatic phase (selected)	Eighth-order astigmatic phase
Quadratic coefficients	c_1x^2	9329.1	9336.5	9565.2	10070 √	10070
	c_2y^2	9329.1	9132.4	8178.1	8099.4 √	8099.6
Quartic coefficients	c_3x^4	--	--	-94.997	-712.23 √	-712.22
	$c_4x^2y^2$	--	--	898.88	403.15 √	389.98
	c_5y^4	--	--	1200.0	1200.0 √	1200.0
Sixth-order coefficients	c_6x^6	--	--	--	260.88 √	260.89
	$c_7x^4y^2$	--	--	--	320.00 √	320.00
	$c_8x^2y^4$	--	--	--	320.00 √	320.00
	c_9y^6	--	--	--	320.00 √	320.00
Eighth-order coefficients	$c_{10}x^8$	--	--	--	--	0.016
	$c_{11}x^6y^2$	--	--	--	--	1.820
	$c_{12}x^4y^4$	--	--	--	--	39.738
	$c_{13}x^2y^6$	--	--	--	--	99.770
	$c_{14}y^8$	--	--	--	--	317.56
FOV		102.720° × 29.374°	102.802° × 28.760°	104.287° × 26.368°	104.618° (*) × 26.304°	104.644° × 26.306°
Sum of merit functions for spot RMS radius (Norm.)		1	0.9914	0.7113	0.6957	0.6932
Weighted sum of all merit functions (Norm.)		1	0.9942	0.6894	0.6607	0.6575
Decrease percentage		0	0.58 %	31.05 %	33.93 %	34.25 %

Notes: The 102° FOV in Fig. **3**c is slightly smaller than the 104.618° reported here, due to the latter being based on the wavelength range [1541.0 nm, 1554.0 nm], whereas the actual edge channel wavelengths are [1541.7 nm, 1553.3 nm].

Based on the data in Table S3, introducing quartic astigmatic terms (x^4, x^2y^2, y^4) reduces the "Sum of merit functions for spot RMS radius" by ~30%, indicating a significant decrease in spot size via astigmatism suppression. Sixth-order terms refine the wavefront and yield gains of ~3% compared to the fourth-order configuration. Extending the phase to the eighth-order provides only ~0.3% improvement relative to the sixth-order phase, which suggests that sixth-order terms already capture the dominant astigmatic correction requirements. Additionally, our AML relies on 8-level phase

quantization for meta-atoms; higher-order terms demand finer phase gradients that exceed the resolution of 8-level quantization, introducing fabrication errors that negate theoretical performance gains. Thus, we ultimately chose to limit the phase to the sixth-order to balance performance, fabrication feasibility, and model simplicity.

(iii) Optimized results: The final optimized phase profile of the AML is shown in Fig. **3**b of the main text. Based on this phase profile, we obtained the final ray tracing results, as shown in Fig. **S4**b(i). The inset of this figure shows the spatial distribution of the output beams after passing through the AML, while the corresponding output angles $\Theta_{x\mathrm{AML}}$ and $\Theta_{y\mathrm{AML}}$ are presented in Fig. **3**c of the main text. For three wavelengths—1541.0 nm (shortest), 1547.5 nm (central), and 1554.0 nm (longest)—the spot diagrams of the output beams at different FOVs are shown in Fig. **S4**b(ii). This figure reveals that most spots across the entire FOV are distributed within the Airy disk defined by the system's diffraction limit (with values of 9.830 mrad, 9.872 mrad, and 9.913 mrad for the three wavelengths, respectively). Slight divergence degradation is observed in extremely peripheral FOVs, which is an acceptable trade-off for wide-FOV imaging—where edge regions are less critical for primary target detection.

(3) Unit cell design

The meta-atom unit cell is a silicon-on-sapphire (SOS) square nanopillar, with the Si nanopillar centered within the cell, as illustrated in Fig. **S4**c(i) and (ii). Fixed structural parameters include the nanopillar height h = 800 nm and square lattice constant p = 651 nm—the former is set to ensure the transmission phase of the unit cell covers a $>2\pi$ range, while the latter satisfies the subwavelength requirement for the operational wavelength ($<\lambda/2$). Using the finite-difference time-domain (FDTD) method implemented in *Lumerical* for simulations, we performed a parameter sweep of the nanopillar width w (ranging from 0.10 μm to 0.55 μm) to obtain the phase response (Φ) and transmittance (T) of the unit cell for different nanopillar sizes. Based on these results, 8 distinct unit cells were selected to cover a complete 2π phase range with uniform spacing of $\pi/4$, as shown in Fig. **S4**c(iii) and (iv). The nanopillar widths w for units No. #1-8 are 222, 268, 293, 313, 332, 354, 382, and 485 nm, respectively. The minimum feature size is 166 nm (calculated as $p - w_{\mathrm{max}}$), which is compatible with standard micro-nano fabrication techniques. Furthermore, based on the parameter sweep curves, Fig. **S4**c(v) depicts the phase responses and transmittances of the 8 designed unit cells when their sizes are uniformly increased or decreased by 10 nm, corresponding to the impact of potential fabrication errors on the unit cell phase. In addition, angular spectrum diffraction simulations show that when nanopillar fabrication errors are -10 nm or +10 nm, they only impose an overall phase offset on the original phase profile, with relatively small additional phase perturbations atop this offset. Such perturbations introduce beam deflection angle errors of 0.34% and -0.57%, respectively, for the beam at the edge FOV (with a ~50° deflection angle in the error-free case). These results demonstrate that our system possesses a certain tolerance to potential fabrication errors.

(4) Simulation verification & micro-nano fabrication

(i) The AML phase distribution obtained in Step (2) underwent 8-level quantization. Combined with the unit cell structural parameters from Step (3), full-wave simulations of several local AML regions were constructed using FDTD to verify beam deflection performance, as illustrated in Fig. **S4**d(i).

(ii) After the above verification, the GDS file for the entire AML was generated, as shown in Fig. **S4**d(ii).

(iii) Micro-nano fabrication of the AML was performed based on the GDS file, with the fabrication flowchart shown in Fig. **S4**d(iii). First, the SOS substrate was cleaned with piranha solution followed by ultrasonic cleaning in acetone and isopropanol. Next, a layer of the electron-beam resist (ma-N2401, Micro Resist Technology) was spin-coated and baked (90°C, 60 s). Then, the metalens pattern was defined via electron-beam lithography (Elionix ELS-F125) before developing in ma-D 525 (room temperature, ~10 s). Finally, inductively coupled plasma etching (SENTECH SI 500) with SF_6/C_4F_8 mixture transferred the pattern to the silicon layer and removed residual resist.

Supplementary Section 5: Characterization of the beam deflection angle and FOV.

5.1 Characterization of beam deflection angle deviations

To validate the expanded FOV capability of the designed AML, we characterized the deviations between the measured and theoretical values of the system's output beam deflection angles:

$$\Delta\Theta_{x,y} = \Theta_{x,y}(\text{meas.}) - \Theta_{x,y}(\text{theo.}) \tag{S7}$$

where $\Theta_{x/y}$ (meas.) and $\Theta_{x/y}$ (theo.) denote the measured and theoretical beam deflection angles in the *x*/*y* directions, respectively. The measured values were obtained by performing far-field spot position measurements at specific scanning positions, where a small reflector was moved to identify the location with the strongest echo signal. From this, the beam's azimuthal angles were calculated relative to the AML. The theoretical values were derived from the beam emission angles obtained through *Zemax* simulations.

For *x*/*y*AOD scanning points of 20 and 83, respectively (corresponding to the scanning mode in Fig. **4**a-d), we measured the output angles Θ_x and Θ_y under varying scanning positions: $N_{x\text{AOD}}$: #1, #4, #7, #9, #12, #14, #17, #20; $N_{y\text{AOD}}$: #1, #21, #42, #63, #83; N_λ: #4, #15, #27. This resulted in a total of 8×5×3×2 datasets. The deviations from theoretical values, $\Delta\Theta_x$ and $\Delta\Theta_y$, are illustrated in Fig. S5a(i)-(iii) and b(i)-(iii). By performing linear fitting on these datasets, we determined the deviations across all scanning points and subsequently corrected the actual beam scanning angles, as shown in Fig. S5a (iv) and b (iv).

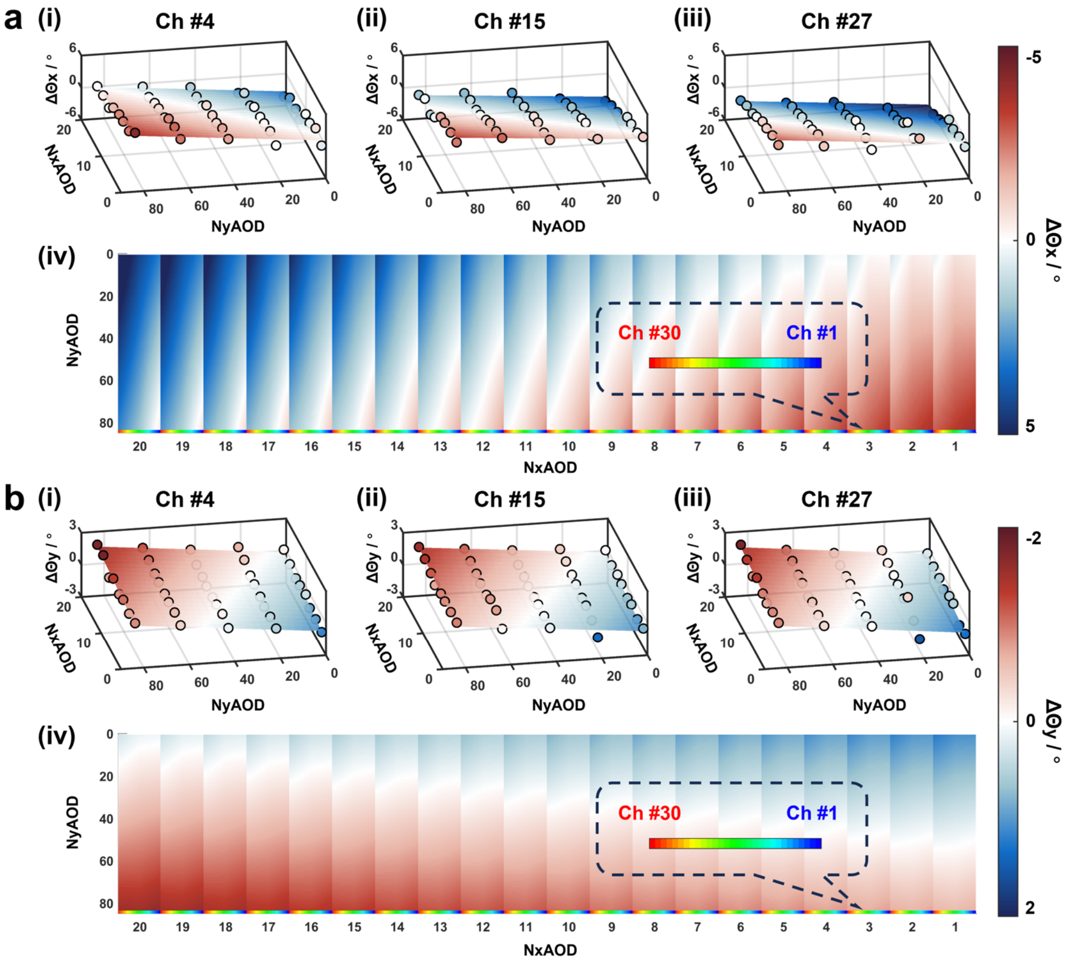

Fig. S5 | Characterization of beam deflection angle deviations. (**a**) $\Delta\Theta_x$ and (**b**) $\Delta\Theta_y$. (i)-(iii) Angular deviations for Ch #4, #15, and #27; (iv) Angular deviations across all scanning points derived from the linear fitting of (i)-(iii). The color bar in the inset indicates that each *x*AOD scanning position includes 30 spectral scanning points (totaling 20 × 30 = 600 scanning points in the *x*-direction).

5.2 Wide-FOV 2D imaging experiment

To characterize the wide-FOV performance of the fabricated AML, we conducted a 2D imaging experiment using a 1D scanning beam. As shown in Fig. **S6**a, four objects with reflective tape are placed at different positions on the same horizontal plane. The horizontal width of the objects and their center positions on the horizontal plane (x_i, z_i) are indicated in Fig. **S6**b, corresponding to angle positions of -47.35°, -9.49°, 27.20°, and 45.74°. The left edge angle of object 1 is -50.33°, while the right edge angle of object 4 is 50.84°, resulting in a required FOV of 101.17°.

We fixed *y*AOD at its center driving frequency of 50 MHz and varied *x*AOD's driving frequency ($\Delta f_{x\text{AOD}}$ = 3 MHz for 7 positions), enabling spectral-AO cascade 1D beam scanning. This produced a series of horizontal scanning spots at Θ_y = 0° as depicted in Fig. **3**c of the main text, with a theoretical maximum FOV range of [-51.30°, 50.96°], covering the edge FOV of the objects at [-50.33°, 50.84°]. In Fig. **S6**c, an orange pulse along with the subsequent blue echoes represents a set of spectral scanning data (with N_λ = 30 angular spatial positions). There are 7 such sets of spectral scanning data, corresponding to 7 scanning points of *x*AOD (scanning towards the negative *x*-direction), resulting in a total of 7×30 angular positions.

As illustrated in the inset of Fig. **S6**c, the TOF information is obtained from the time delay between the echoes and the calibrated reference timing (gray dashed line). This allowed us to derive an intensity-distance mapping at 210 angular coordinates. By converting the polar coordinates to Cartesian coordinates, we were able to reconstruct a 2D image resembling the top view of the objects, as shown in Fig. **S6**d. This confirms that we successfully detected all four objects experimentally, thereby validating our theoretical FOV coverage of the area. Moreover, the spatial positions of the imaged objects closely match the actual measurements (green points and orange lines), further demonstrating the wide-FOV performance of the AML-based LiDAR system.

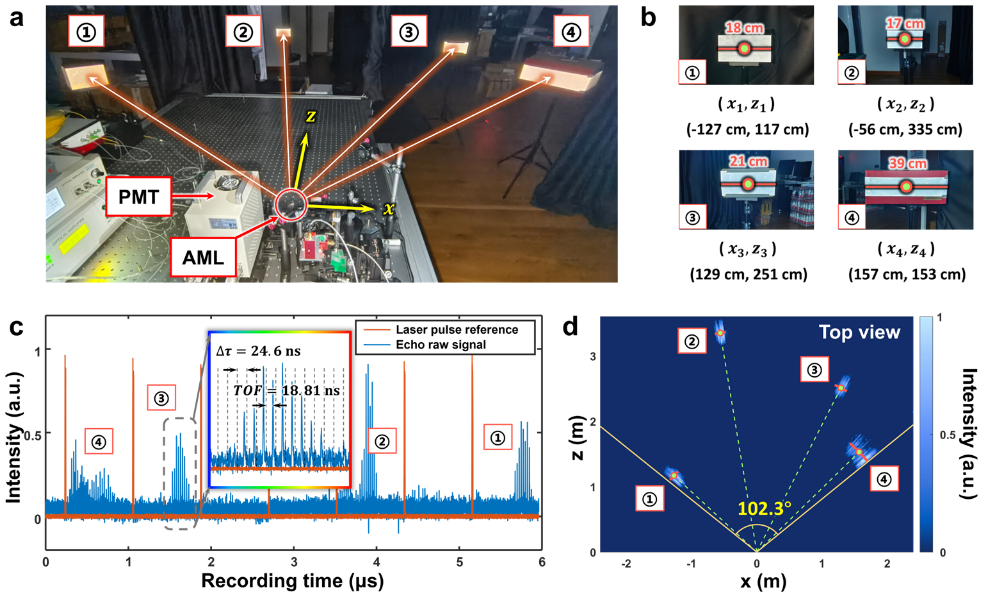

Fig. S6 | Wide-FOV ranging capability. (**a**) Imaging scenario with all objects positioned on the same horizontal plane. (**b**) Four objects covered with reflective tape, with their widths indicated by orange lines and their central positions on the horizontal plane denoted by green dots at coordinates (x_i, z_i). (**c**) Raw echo signal of the scanned objects (blue curve) and reference laser pulse (orange curve) recorded during a single scanning cycle. (**d**) Ranging image of the four objects. Green dashed lines represent the true angular positions calculated from (**b**), while the orange line segments and green dots mark the central positions and widths of the objects. The graph illustrates the system's ability to detect all four objects, achieving a horizontal FOV exceeding 102°.

Our current maximum FOV is not a limit imposed by the AML itself; instead, it is constrained by the periodicity of the meta-atoms, which determines the maximum phase gradient they can provide. However, this periodicity is also limited by the constraints of micro-nano fabrication techniques. Theoretically, we could achieve larger FOV by utilizing a smaller meta-atom periodicity that offers a greater phase gradient.

Supplementary Section 6: Characterization of the beam divergence angle.

To characterize the astigmatism correction capability of the AML for practical beam conditions, we used a CCD (Allied Vision, Goldeye G-130 TEC1, 1280 × 1024 pixels, 5 µm × 5 µm pixel size) to record the intensity distribution of the beam as it propagated downstream of the collimator (COL) (representing the input of the entire optical system), the COL + BG, and the AML (whole system). From these measurements, we fitted the divergence and deflection angles of the beams using the following Gaussian function model:

$$I_{gaussian\_fitted}\ (x,y)=A\cdot\exp(-\frac{(x-x_0)^2}{2\sigma_{\omega x}{}^2}-\frac{(y-y_0)^2}{2\sigma_{\omega y}{}^2})+I_n \quad (S8)$$

$$\sigma_{\omega x}=\frac{1}{2}\omega_x\ ,\ \sigma_{\omega y}=\frac{1}{2}\omega_y \quad (S9)$$

where I_n represents the background noise intensity, (x, y) are the 2D coordinates on the CCD, (x_0, y_0) are the Gaussian beam center positions, and ω_x, ω_y represent the beam waist radii in the x/y-directions (the radius at which the intensity falls to e^{-2} of its maximum). The beam divergence angle $\theta_{x,y}$ is calculated by $\boldsymbol{d}\omega_{x,y}/\boldsymbol{dz}$, where z denotes the propagation distance.

Figure S7 shows the beam propagation evolution downstream of the COL. After fitting, the input divergence angles θ_x and θ_y were found to be 0.803 mrad and 0.790 mrad, respectively, indicating an almost perfectly circularly symmetric Gaussian beam (with an average value of ~0.80 mrad taken as the initial beam divergence angle).

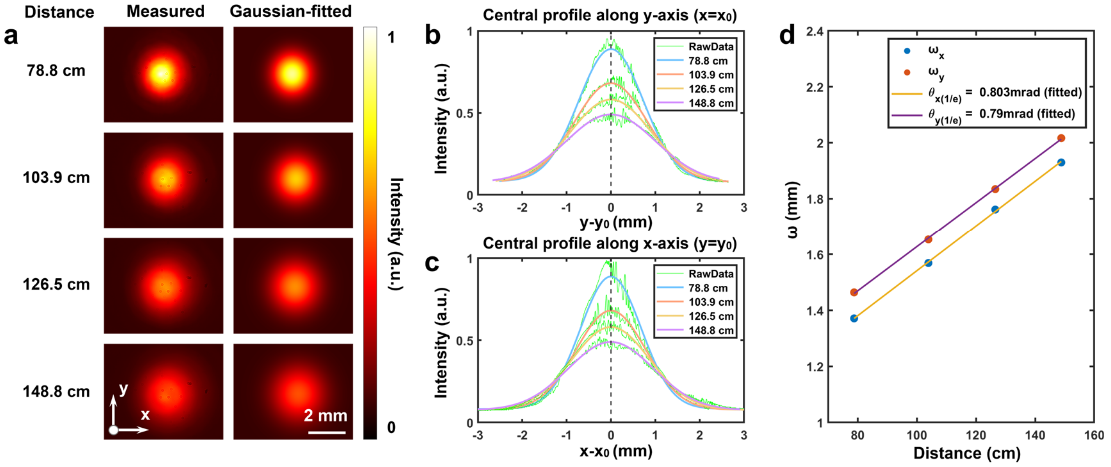

Fig. S7 | Beam propagation evolution after the collimator (COL). (**a**) Beam intensity distributions at different distances downstream of the COL (Left: measured; Right: Gaussian-fitted). (**b**)(**c**) Central intensity profiles of the beam along (**b**) the y-axis ($x = x_0$) and (**c**) the x-axis ($y = y_0$). (**d**) Beam waist radii ω_x, ω_y as functions of propagation distance, and the corresponding divergence angles θ_x, θ_y derived from linear fitting.

Figure S8 shows the beam propagation evolution downstream of the COL and the BG. We fitted the deflection angles Θ induced by grating dispersion based on the variation of the beam's center positions x_0 at different wavelengths (taking the center of the middle wavelength beam as the zero reference, calculated by $d(x_{0\_\lambda i} - x_{0\_\lambda 21})/dz$), as shown in Fig. S8b. Through fitting, the average angular separation between adjacent wavelengths was found to be ~ 0.56 mrad. Additionally, the average beam divergence angles θ_x and θ_y were ~2.36 mrad and ~0.8 mrad, respectively, indicating significant astigmatic properties (acting as the data source of the x/y-direction divergence angles without ML in Table **1**).

In comparison, Fig. S9 shows the beam propagation evolution downstream of the AML (whole system). Fig. S9b shows that the average angular separation between adjacent wavelengths is ~9.4 mrad. The fitted average beam divergence angles θ_x and θ_y were 11.8 mrad and 10.2 mrad, respectively, with significant suppression of astigmatism. Furthermore,

when compared with the simulated Airy disk size (~9.9 mrad) in Fig. S4b(ii), this result also demonstrates that the system delivers near-diffraction-limited performance. Moreover, we used the ratio of the deflection angle between adjacent wavelengths to the divergence angle of a single wavelength to describe the relative beam separation:

$$\frac{\overline{\Delta\Theta_{\lambda_i-\lambda_{i+1}}}}{\overline{\theta_x}}(\text{before astigmatism correction})=\frac{0.56\ \text{mrad}}{2.36\ \text{mrad}}=23.7\% \tag{S10}$$

$$\frac{\overline{\Delta\Theta_{\lambda_i-\lambda_{i+1}}}}{\overline{\theta_x}}(\text{after astigmatism correction})=\frac{9.4\ \text{mrad}}{11.8\ \text{mrad}}=79.7\% \tag{S11}$$

It is evident that after the AML corrects the astigmatism, the beam separation between adjacent spectral channels increases (~3.4-fold improvement), which is not achievable with conventional non-astigmatic telescopic systems that simultaneously amplify both the deflection and divergence angles.

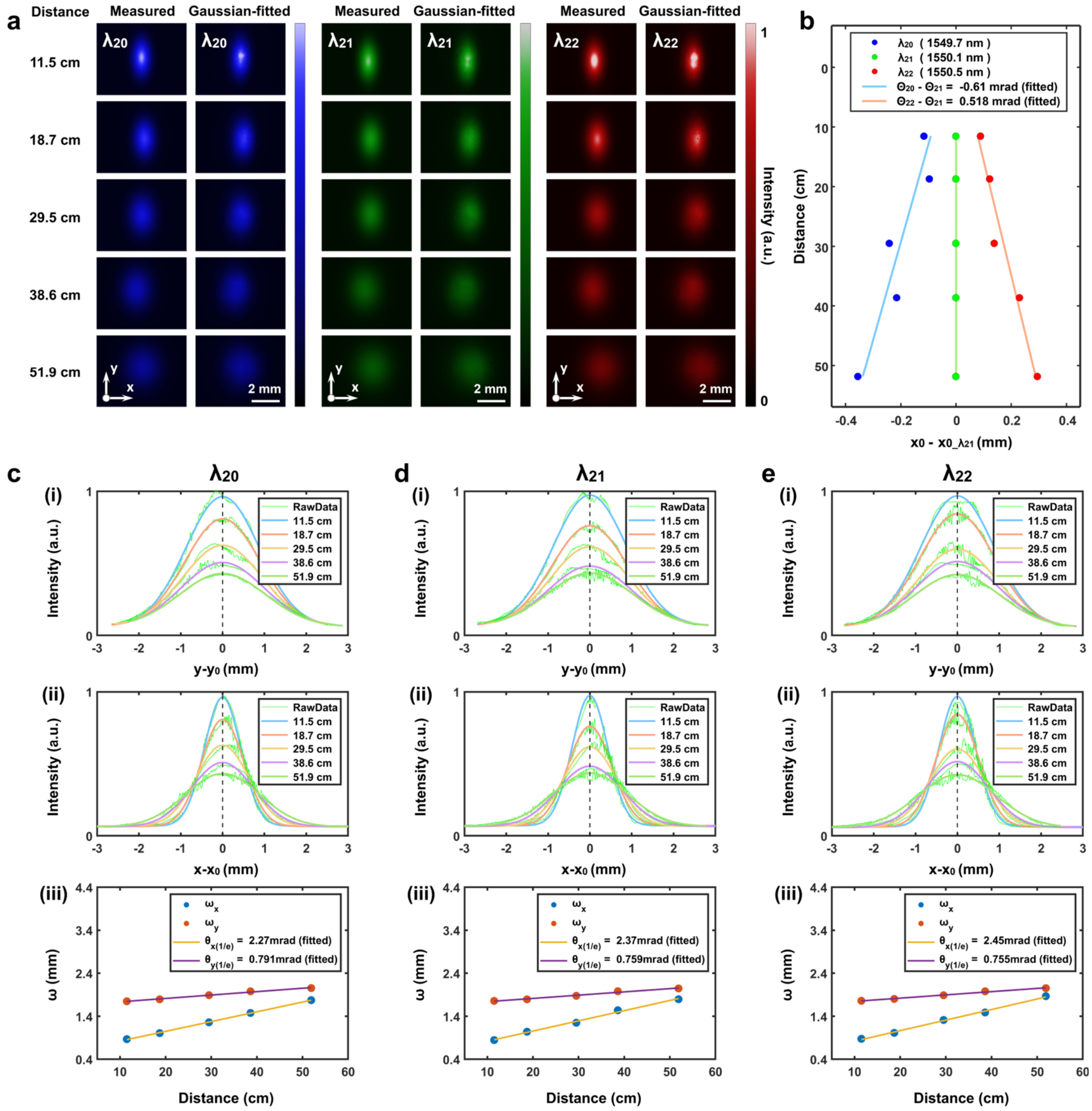

Fig. S8 | Beam propagation evolution after the COL and BG. (**a**) Beam intensity distributions for three adjacent spectral channels (λ_{20}, λ_{21}, λ_{22}) at different distances downstream of the COL and BG. (**b**) Relative angular deviations of the three-channel beams obtained from the center positions x_0 of their intensity distributions. (**c**)-(**e**) Central intensity profiles of the beam along (i) the y-axis ($x = x_0$) and (ii) the x-axis ($y = y_0$). (iii) Beam waist radii ω_x, ω_y as functions of propagation distance, and the corresponding divergence angles θ_x, θ_y derived from linear fitting (average: ~2.36 mrad *vs*. ~0.8 mrad). Panels (**c**), (**d**), and (**e**) correspond to λ_{20}, λ_{21}, λ_{22}, respectively.

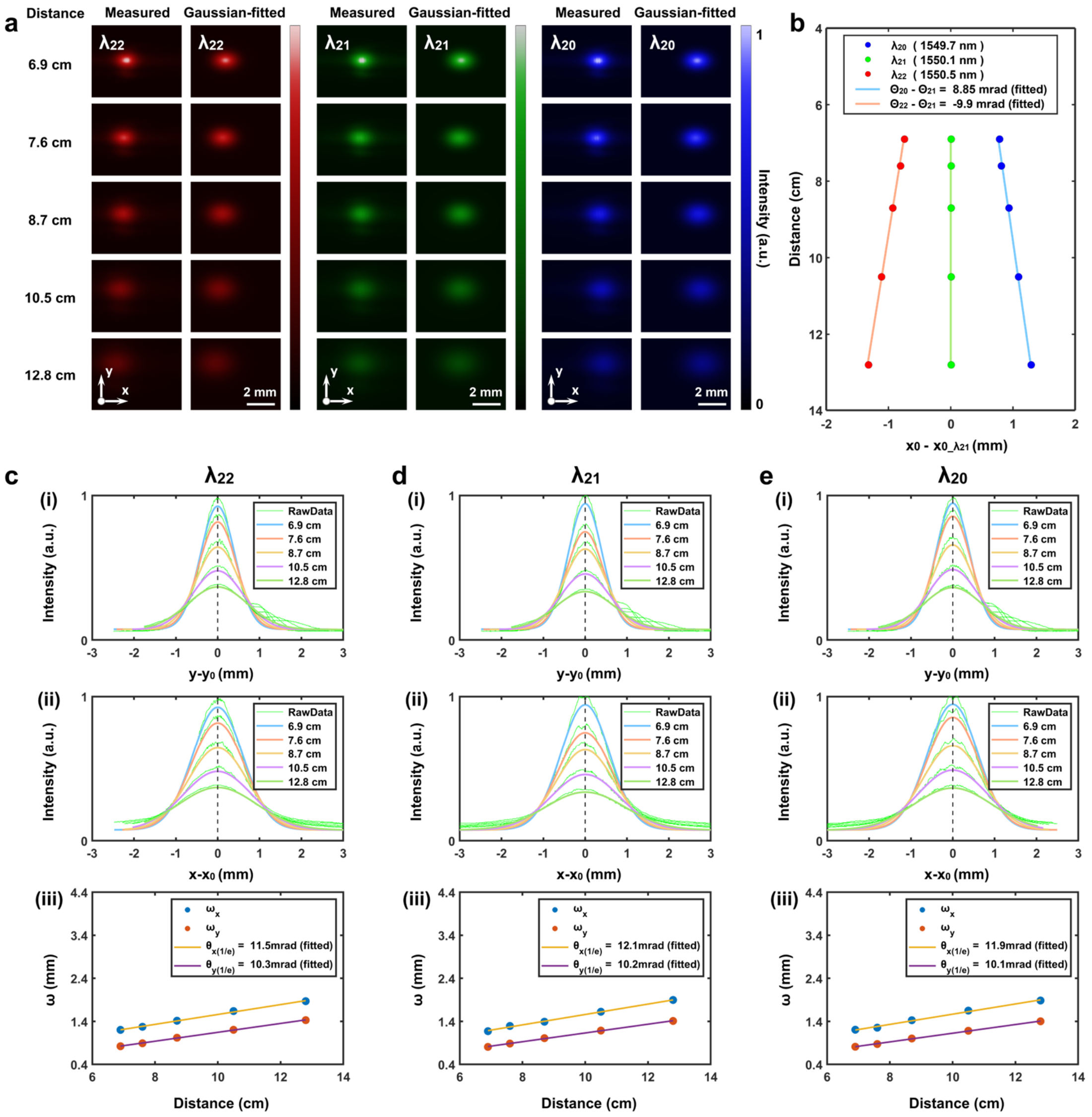

Fig. S9 | Beam propagation evolution after the AML (whole system). (**a**) Beam intensity distributions for three adjacent spectral channels (λ_{20}, λ_{21}, λ_{22}) at different distances downstream of the AML. (**b**) Relative angular deviations of the three-channel beams obtained from the center positions x_0 of their intensity distributions. (**c**)-(**e**) Central intensity profiles of the beam along (i) the y-axis ($x = x_0$) and (ii) the x-axis ($y = y_0$). (iii) Beam waist radii ω_x, ω_y as functions of propagation distance, and the corresponding divergence angles θ_x, θ_y derived from linear fitting (average: 11.8 mrad *vs*. 10.2 mrad). Panels (**c**), (**d**), and (**e**) correspond to λ_{22}, λ_{21}, λ_{20}, respectively.

Supplementary Section 7: Data processing procedure.

This work employs two data processing methods to reconstruct 3D information from the measured LiDAR raw echo signals, as illustrated in Fig. S10.

The first method directly extracts the TOF corresponding to the echo peak of each pulse to generate a point cloud (Fig. **4** of the main text). We employ a streamlined, general workflow consisting of "signal preprocessing → TOF calculation → angular position matching → point cloud reconstruction," with detailed steps as follows:

(1) Signal preprocessing for noise-suppression: As depicted in Fig. S10a, we begin by applying a series of noise-suppression procedures to the raw echoes, including β-fold temporal averaging (if $\beta > 1$), frequency-domain low-pass filtering, and amplitude-threshold-based denoising.

(2) TOF extraction and distance calculation: The TOF is determined by the time delay between the echo signal and the calibrated reference timing, as shown in Fig. S10a(i)(ii) (the calibration process is detailed in Supplementary Section 2). Then, a three-point fitting method[11] is used to further refine TOF estimation and the target distance D is calculated via $D = c\cdot\text{TOF}/2$.

(3) Angular position matching: Combining the scanning timing synchronization signal and the scanning angle calibration in Fig. S5, we map each scanning point to its corresponding angular coordinates (Θ_x, Θ_y). This ensures that each distance value D is accurately associated with its spatial angular position.

(4) 3D point cloud reconstruction and optimization: Using the angular coordinates (Θ_x, Θ_y) and distance D, we obtain the corresponding 3D point clouds. This is followed by a statistical filtering step (via *Matlab*'s *pcdenoise* function) to remove outliers and generate a clearer point cloud reconstruction, as shown in Fig. S10b.

Rather than assigning a single TOF to each pulse, the second method accounts for beam divergence and integrates all echo signals to perform slice-based reconstruction across the entire 3D volume (Fig. **5** of the main text). As depicted in Fig. S10c, subpixel reconstruction integrates the Gaussian intensity distribution of the beams within the pixel grid (Θ_{xm}, Θ_{yn}, D_k). Each pixel's intensity is weighted by contributions from all nearby beams, as defined in the following equation:

$$I_{\text{reconstructed}}(\Theta_{xm},\Theta_{yn},D_k)=\frac{\sum_i\sum_j I(\Theta_{xi},\Theta_{yi},D_k)\cdot\exp\left(-\frac{(\Theta_{xi}-\Theta_{xm})^2}{2\sigma_x^2}-\frac{(\Theta_{yi}-\Theta_{yn})^2}{2\sigma_y^2}\right)}{\sum_i\sum_j 1\cdot\exp\left(-\frac{(\Theta_{xi}-\Theta_{xm})^2}{2\sigma_x^2}-\frac{(\Theta_{yi}-\Theta_{yn})^2}{2\sigma_y^2}\right)} \tag{S12}$$

$$\sigma_x=\frac{1}{2}\theta_x\ ,\ \sigma_y=\frac{1}{2}\theta_y \tag{S13}$$

where I (Θ_{xi}, Θ_{yj}, D_k) denotes the echo signal intensity at the i-th and j-th actual beam scanning positions in the x- and y-directions, respectively, and the k-th distance (corresponding to a specific echo timestamp). Meanwhile, $I_{\text{reconstructed}}$ (Θ_{xm}, Θ_{yn}, D_k) represents the reconstructed intensity at the m/n-th grid position in the x/y-direction, and the k-th distance. The numerator on the right-hand side of Eq. S12 accounts for the combined influence of all nearby beams (Θ_{xi}, Θ_{yj}) on the grid position (Θ_{xm}, Θ_{yn}), while the denominator corrects for the non-uniform distribution of the actual beams. The Gaussian beam's standard deviation, $\sigma_{x,y}$, is defined as half of the divergence angle $\theta_{x,y}$ at which the beam intensity falls to e^{-2} of its maximum.

As shown in Fig. S10c(iii), the final reconstructed intensity $I_{\text{reconstructed}}$ (Θ_x, Θ_y, D) can be visualized as a series of distance slices, revealing fine spatial details that are difficult to resolve using direct point-cloud processing (Fig. **5** in the main text).

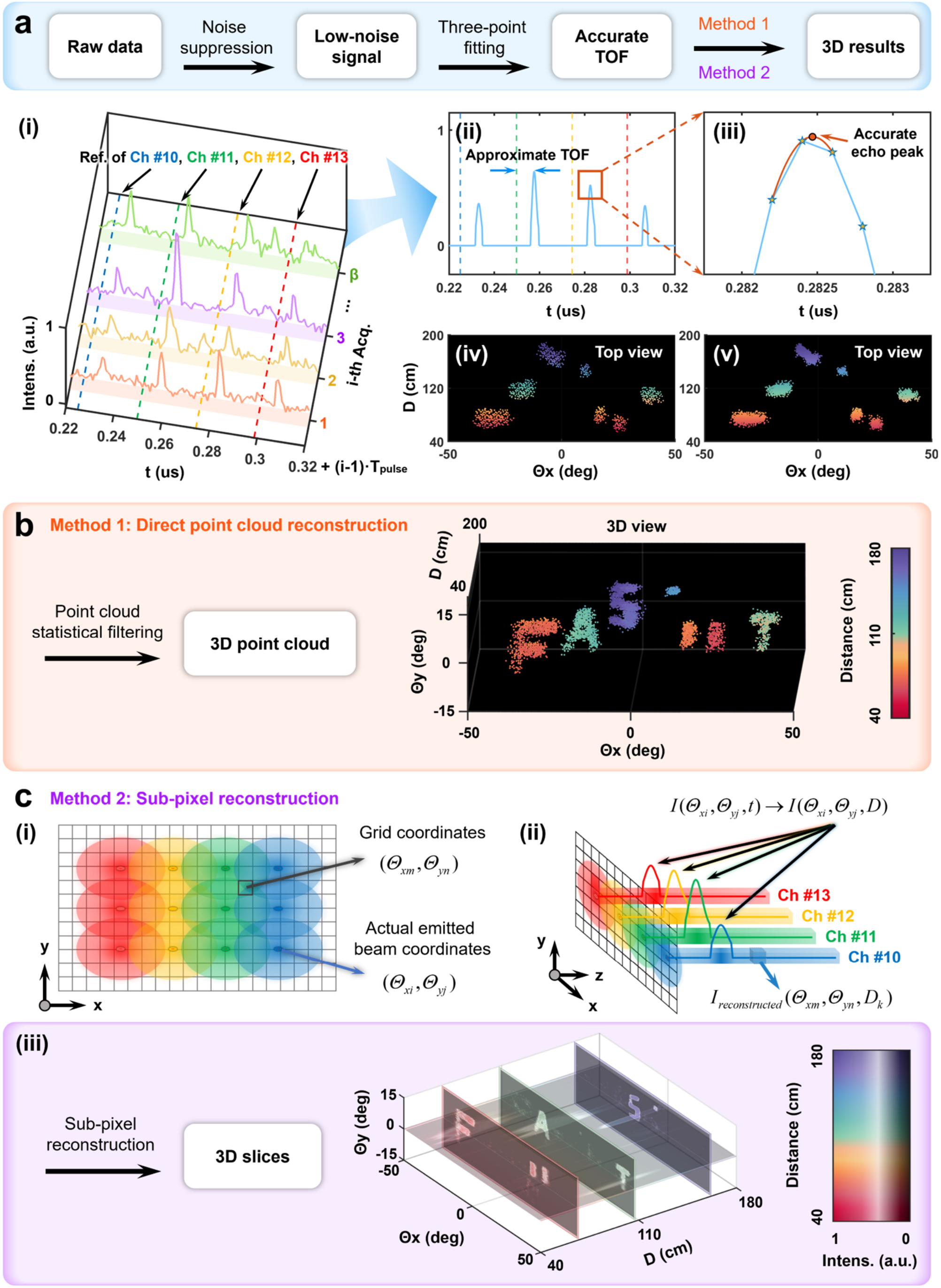

Fig. S10 | Data processing for 3D reconstruction. (**a**) Flowchart of the raw echo data processing procedure: (i) β repeated acquisitions at a single scanning position. (ii) Apply multiple averaging steps, frequency-domain filtering, and threshold-based denoising to obtain a low-noise signal. (iii) Use three-point fitting to extract more accurate TOF. (iv)&(v) Top view images of the reconstruction results before and after three-point fitting, highlighting a substantial improvement in ranging resolution. (**b**) Method 1: Direct point cloud reconstruction to generate the final 3D point cloud image. (**c**) Method 2: Subpixel reconstruction. (i) In method 1, every pulse is mapped to a point (Θ_{xi}, Θ_{yj}, D_{peak}) based on the timestamp of the echo peak. In contrast, subpixel reconstruction accounts for beam divergence: within the pixel grid (Θ_{xm}, Θ_{yn}), the intensity is no longer binary ("1 or 0") but is modulated by the Gaussian distribution of surrounding beams. (ii) From each actual emitted beam, the entire echo waveform is used to obtain I (Θ_{xi}, Θ_{yj}, D), enabling further 3D reconstruction of $I_{\mathrm{reconstructed}}$ (Θ_{xm}, Θ_{yn}, D_k) for every grid location. (iii) The resulting $I_{\mathrm{reconstructed}}$ can be visualized as multiple slice views, revealing the intensity distribution at various distances.

Supplementary Section 8: Discussion on ranging resolution.

The achieved ranging/depth resolution is determined by the system's timing precision, which is affected by several key factors: (i) the stability of the laser source's pulse period (the timing deviations of the laser source pulses have a standard deviation of ~30 ps); (ii) the laser source's pulse width (~100 ps); (iii) pulse broadening introduced by modulation devices, (iv) the inherent timing jitter of the detector; and (v) the sampling rate of the digitizer. In our experimental setup, the primary contributors to timing error are factors (iv) and (v).

On the one hand, the intrinsic error of the photomultiplier tube (PMT) arises from the transit time caused by its multi-stage amplification mechanism. When a single photon strikes the PMT, the photocathode converts it into photoelectrons, which are then multiplied through a series of dynodes before reaching the anode. The total transit time of the electron group depends on each incident photon, resulting in a dispersion known as transit time spread (TTS). According to the manufacturer's specifications, the PMT (HAMAMATSU, H10330C-75) has a TTS of ~0.4 ns.

On the other hand, in the experiments presented in Figs. **4** and **5** of the main text, we utilize a data acquisition card (Teledyne, ADQ7DC) as the digitizer (analog-to-digital converter, ADC), which records data at a sampling rate of 5 GS/s, yielding a time resolution of 0.2 ns.

As a result, the overall ranging resolution of our system is primarily constrained by the PMT's TTS of ~0.4 ns, which corresponds to a theoretical ranging resolution of ~6 cm. However, as shown in Fig. **4**b of the main text, we actually achieve an improved ranging standard deviation σ of ~3 cm. This enhancement is primarily attributed to the use of three-point fitting (Supplementary Section 7), which enables sub-sample TOF estimation. In addition, the measured σ reflects the statistical variation of echo returns from a relatively uniform target surface, rather than the intrinsic timing jitter associated with individual photon detection events.

Furthermore, it is important to emphasize that our proposed method does not impose strict constraints on detector selection. In other words, the ranging precision is not limited by the proposed method itself; we simply opted for a PMT in our experiments. If a detector with higher timing precision (e.g., a higher-bandwidth avalanche photodiode, APD) is used, the ranging resolution can be further improved.

Supplementary Section 9: Power loss analysis & discussion on long-range detection.

As shown in Fig. S11, the primary power losses in our system occur at the AOD and the AML, mainly due to fabrication defects. Consequently, the actual effective power at the transmitter end is only a few milliwatts (~2 mW after the AML), limiting the detection range of our imaging experiments, which were conducted at distances of < 3.4 m indoors. According to the inverse square law governing the relationship between received scattered power and detection distance, achieving longer-range detection requires addressing the challenge of weak echo signals. This can be approached through several strategies: (i) increasing the transmission power (with attention to the devices' damage threshold); (ii) further suppressing angular divergence; (iii) employing more sensitive detectors; and (iv) performing frequency up-conversion on the echo signals to shift them into the visible spectrum, aligning within the high responsivity range of mature silicon-based photodetectors, thus improving detection efficiency[12, 13].

a b

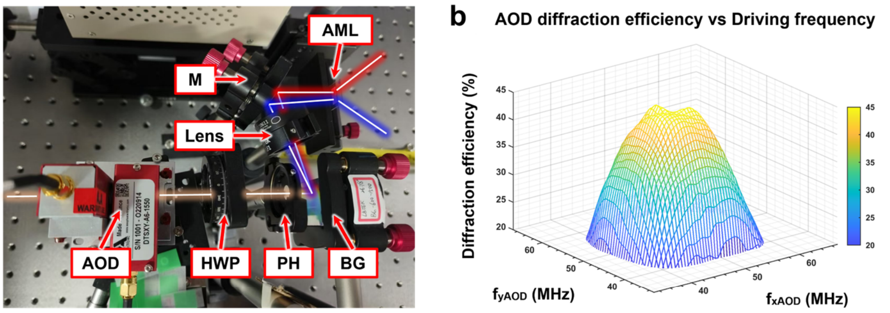

c

	HWP	BG	Lens	M	AML (1 order)	AML (0 order)	AML (Total)
T_{min}	97.1%	69.3%	97.4%	97.0%	32.3%	13.3%	46.2%
T_{max}	99.2%	88.9%	99.0%	99.9%	51.5%	20.6%	65.2%
$\bar{T}$ (Loss)	98.8% (0.05 dB)	83.0% (0.81 dB)	92.6% (0.33 dB)	98.1% (0.08 dB)	40.1% (3.97 dB)	15.2%	55.3%

Fig. S11 | Power loss analysis. (a) Photograph of the optical system at the transmitter end, after the COL. (**b**) Diffraction efficiency of the dual-axis AOD. (**c**) Transmittance/diffraction efficiency of other optical components (including a total of 50 measurements evenly distributed across the entire FOV, with the last row representing the average result after removing outliers).

Supplementary Section 10: Maximum detectable depth span (Δd_m) and ambiguity distance (d_m).

To better clarify the two parameters Δd_m and d_m mentioned in the *Discussion* section of the main text, we refer to the echo recognition results under different target distances and depths (as illustrated in Fig. **S12**). These results enable a more intuitive distinction between the following two core distance parameters:

(1) Maximum detectable depth span (Δd_m): It denotes the upper limit of the target depth difference that the system can detect unambiguously (determined by the sub-pulses temporal interval $\Delta\tau$, with the formula $\Delta d_m = c{\cdot}\Delta\tau/2$, where c is the speed of light). Specifically, if the depth difference of detected targets is $\le \Delta d_m$, their echoes can be clearly distinguished by each spectral channel (Fig. **S12**a); if the depth difference exceeds Δd_m, the echoes will go beyond the time window of the current channel and enter the detection window of the next spectral channel, leading to recognition failure (as shown in Fig. **S12**b).

(2) Ambiguity distance (d_m): It represents the upper limit of the absolute target distance that the system can detect unambiguously (determined by the laser pulse period T_{pulse}, with the formula $d_m = c{\cdot}T_{pulse}/2$). If a target's absolute distance exceeds d_m, its echoes will enter the recognition region of the next laser pulse cycle, triggering recognition failure—which aligns with the original definition of the ambiguity distance (as shown in Fig. **S12**d); in contrast, when the target distance lies within the range $\Delta d_m <$ Distance(obj) $\le d_m$ and the depth difference is $\le \Delta d_m$, echoes can be correctly correlated by subsequent spectral channels (via time-stretched sub-pulse windows), thus enabling unambiguous detection (as depicted in Fig. **S12**c).

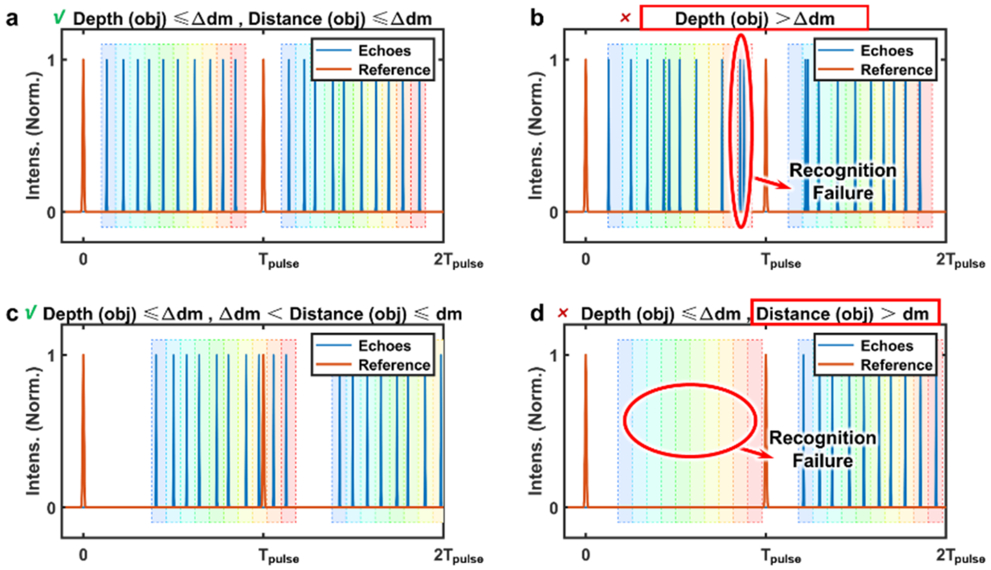

Fig. S12 | Echo recognition results under different target distances and depths (taking 10 spectral channels as an example). (a) Depth(obj) $\le \Delta d_m$, Distance(obj) $\le \Delta d_m$. (**b**) Depth(obj) $> \Delta d_m$. (**c**) Depth(obj) $\le \Delta d_m$, $\Delta d_m <$ Distance(obj) $\le d_m$. (**d**) Depth(obj) $\le \Delta d_m$, Distance(obj) $> d_m$.

References

1. Jiang, Y, Karpf, S, Jalali, B. Time-stretch LiDAR as a spectrally scanned time-of-flight ranging camera. *Nature Photonics* **14**, 14-18 (2020).
2. Juliano Martins, R. et al. Metasurface-enhanced light detection and ranging technology. *Nature Communications* **13**, 5724 (2022).
3. Zang, Z. et al. Ultrafast parallel single-pixel LiDAR with all-optical spectro-temporal encoding. *APL Photonics* **7**, 046102 (2022).
4. Qian, R. et al. Video-rate high-precision time-frequency multiplexed 3D coherent ranging. *Nature Communications* **13**, 1476 (2022).
5. Chen, R. et al. Breaking the temporal and frequency congestion of LiDAR by parallel chaos. *Nature Photonics* **17**, 306-314 (2023).
6. Lukashchuk, A. et al. Dual chirped microcomb based parallel ranging at megapixel-line rates. *Nature Communications* **13**, 3280 (2022).
7. Römer, G, Bechtold, P. Electro-optic and acousto-optic laser beam scanners. *Physics Procedia* **56**, 29-39 (2014).
8. Pu, M. et al. Nanoapertures with ordered rotations: symmetry transformation and wide-angle flat lensing. *Optics Express* **25**, 31471-31477 (2017).
9. Zhang, F. et al. Extreme-angle silicon infrared optics enabled by streamlined surfaces. *Advanced Materials* **33**, 2008157 (2021).
10. Guo, Y. et al. High-efficiency and wide-angle beam steering based on catenary optical fields in ultrathin metalens. *Advanced Optical Materials* **6**, 1800592 (2018).
11. Wang, J. et al. Long-distance ranging with high precision using a soliton microcomb. *Photonics Research* **8**, 1964-1972 (2020).
12. Albota, MA, Wong, FN. Efficient single-photon counting at 1.55 μm by means of frequency upconversion. *Optics Letters* **29**, 1449-1451 (2004).
13. Rehain, P. et al. Noise-tolerant single photon sensitive three-dimensional imager. *Nature Communications* **11**, 921 (2020).
